# Brain network analysis of interictal epileptiform discharges from ECoG to identify epileptogenic zone in pediatric patients with epilepsy and focal cortical dysplasia type II: A retrospective study

**DOI:** 10.3389/fneur.2022.901633

**Published:** 2022-08-05

**Authors:** Zhi Ji Wang, Byoung Ho Noh, Eun Seong Kim, Donghwa Yang, Shan Yang, Nam Young Kim, Yun Jung Hur, Heung Dong Kim

**Affiliations:** ^1^Division of Pediatric Neurology, Department of Pediatrics, Severance Children's Hospital, Epilepsy Research Institute, Yonsei University College of Medicine, Seoul, South Korea; ^2^Radio Frequency Integrated Circuit (RFIC), Kwangwoon University, Seoul, South Korea; ^3^Department of Pediatrics, Kangwon National University Hospital, Chuncheon-si, South Korea; ^4^Division of Pediatric Neurology, Department of Pediatrics, National Health Insurance Service Ilsan Hospital, Goyang-si, South Korea; ^5^Department of Pediatrics, Haeundae Paik Hospital, Inje University College of Medicine, Busan, South Korea

**Keywords:** identification of epileptogenic zone, graph theory analysis, phase transfer entropy, power spectrum compensation, time-frequency analysis

## Abstract

**Objective:**

For patients with drug–resistant focal epilepsy, intracranial monitoring remains the gold standard for surgical intervention. Focal cortical dysplasia (FCD) is the most common cause of pharmacoresistant focal epilepsy in pediatric patients who usually develop seizures in early childhood. Timely removal of the epileptogenic zone (EZ) is necessary to achieve lasting seizure freedom and favorable developmental and cognitive outcomes to improve the quality of life. We applied brain network analysis to investigate potential biomarkers for the diagnosis of EZ that will aid in the resection for pediatric focal epilepsy patients with FCD type II.

**Methods:**

Ten pediatric patients with focal epilepsy diagnosed as FCD type II and that had a follow–up after resection surgery (Engel class I [*n* = 9] and Engel class II [*n* = 1]) were retrospectively included. Time–frequency analysis of phase transfer entropy, graph theory analysis, and power spectrum compensation were combined to calculate brain network parameters based on interictal epileptiform discharges from ECoG.

**Results:**

Clustering coefficient, local efficiency, node out–degree, and node out–strength with higher values are the most reliable biomarkers for the delineation of EZ, and the differences between EZ and margin zone (MZ), and EZ and normal zone (NZ) were significant (*p* < 0.05; Mann–Whitney *U*-test, two–tailed). In particular, the difference between MZ and NZ was significant for patients with frontal FCD (MZ > NZ; *p* < 0.05) but was not significant for patients with extra–frontal FCD.

**Conclusions:**

Brain network analysis, based on the combination of time–frequency analysis of phase transfer entropy, graph theory analysis, and power spectrum compensation, can aid in the diagnosis of EZ for pediatric focal epilepsy patients with FCD type II.

## Introduction

Focal cortical dysplasia (FCD), a subgroup of malformations in cortical development, is characterized by dysmorphic neurons, cortical dyslamination, and differentiation. FCD type II is usually seen in children and is the most common cause of intractable focal epilepsy. In pharmacoresistant focal epilepsy, resection surgery is the most reliable treatment after delineating cerebral areas responsible for seizure generation (1). Ictal patterns are usually associated with the seizure onset zone (SOZ), while interictal epileptiform discharges (IEDs)—generated by a population of neurons in a synchronous state—are a reliable indicator of *in vivo* epileptic tissue. Multiple previous studies have focused on network analysis of ictal and pre–/post–ictal events; however, there are limitations. Determination of resection margins of SOZ from ECoG data alone is not sufficient to achieve seizure freedom (2) and some patients may not have seizure activity during the recording period, making it difficult for the event selection.

IEDs feature a high signal–to–noise ratio and less contamination produced by artifacts, as is commonly seen during ictal events, and are easily captured during electroencephalography (EEG) recording sessions (3, 4). IEDs consist of spike or sharp wave discharges, abnormal slowing waves of the background EEG signal, and high–frequency oscillations (HFOs) (5). HFOs are a promising biomarker for the diagnosis of the epileptogenic zone (EZ), potentially by improving the surgical success of patients with pharmacoresistant epilepsy without the need to record seizures. However, a recent multicenter study revealed that EZ was not correctly identified by HFOs in 31% of patients (6). Roehri et al. found that HFOs or any variants were not statistically better biomarkers for EZ than IED spikes (7). Thus, additional efforts are required to evaluate clinical importance of HFOs based on prospective clinical trials (8). Abnormally slow waves are usually markers of lesion area. Interference can be introduced during electrode insertion or when the electrode is inserted near the resection cavity, causing iatrogenic artifacts (5). Spike or sharp wave discharges are conventional markers of epilepsy that localize the EZ to facilitate targeted surgery (9). In a quantitative iEEG study, 56% patients had a good concordance between spike density and SOZ; a higher rate (75%) was observed in patients with FCD (5).

EZ can be adequately localized by non–invasive multi–modal studies such as the semiology of the signs and symptoms of ictal events, neurophysiological examination, magnetic resonance imaging (MRI), single–photon emission computed tomography (SPECT), subtraction ictal SPECT coregistered to MRI, positron emission tomography (PET), scalp video EEG, etc. (10). However, when the findings regarding the delineation of the region responsible for seizure generation are inconclusive or when the SOZ cannot be precisely localized non–invasively, ECoG should be considered (11). The most common approach for the diagnosis of EZ is visually inspecting the ECoG, however, it is time–consuming and requires a specifically trained neurophysiologist. More sophisticated methods should be further investigated to aid in the localization of EZ because of the high rate of failure in resection surgery, especially for extra–temporal epilepsy patients. Recently, a new interictal marker for seizure localization called source–sink connectivity was applied on 65 adult patients using sEEG. The source–sink metrics (SSMs) predicted outcomes with an accuracy of 79%, much higher than the clinicians' prediction. Additionally, they identified the brain regions with high SSMs were untreated in the failed outcomes (12).

The anatomical structure of the brain supports dynamic neuronal oscillation—a physiological activity that can cross several distinct brain regions and build up a functional network (13), which can be extracted, derived, and statistically evaluated based on time–series data (14), such as EEG. This contributes to the understanding of the organization of the human brain network and makes it possible to diagnose EZ (4) since epilepsy is a network disease.

In this study, brain network analysis, based on a combination of time–frequency analysis of phase transfer entropy, graph theory analysis, and power spectrum compensation, was used for the identification of EZ for pediatric focal epilepsy patients with FCD type II. Additionally, several brain network parameters were calculated to explore potential biomarkers to differentiate EZ, MZ, and NZ.

## Materials and methods

### Patients

**Figure 1:**
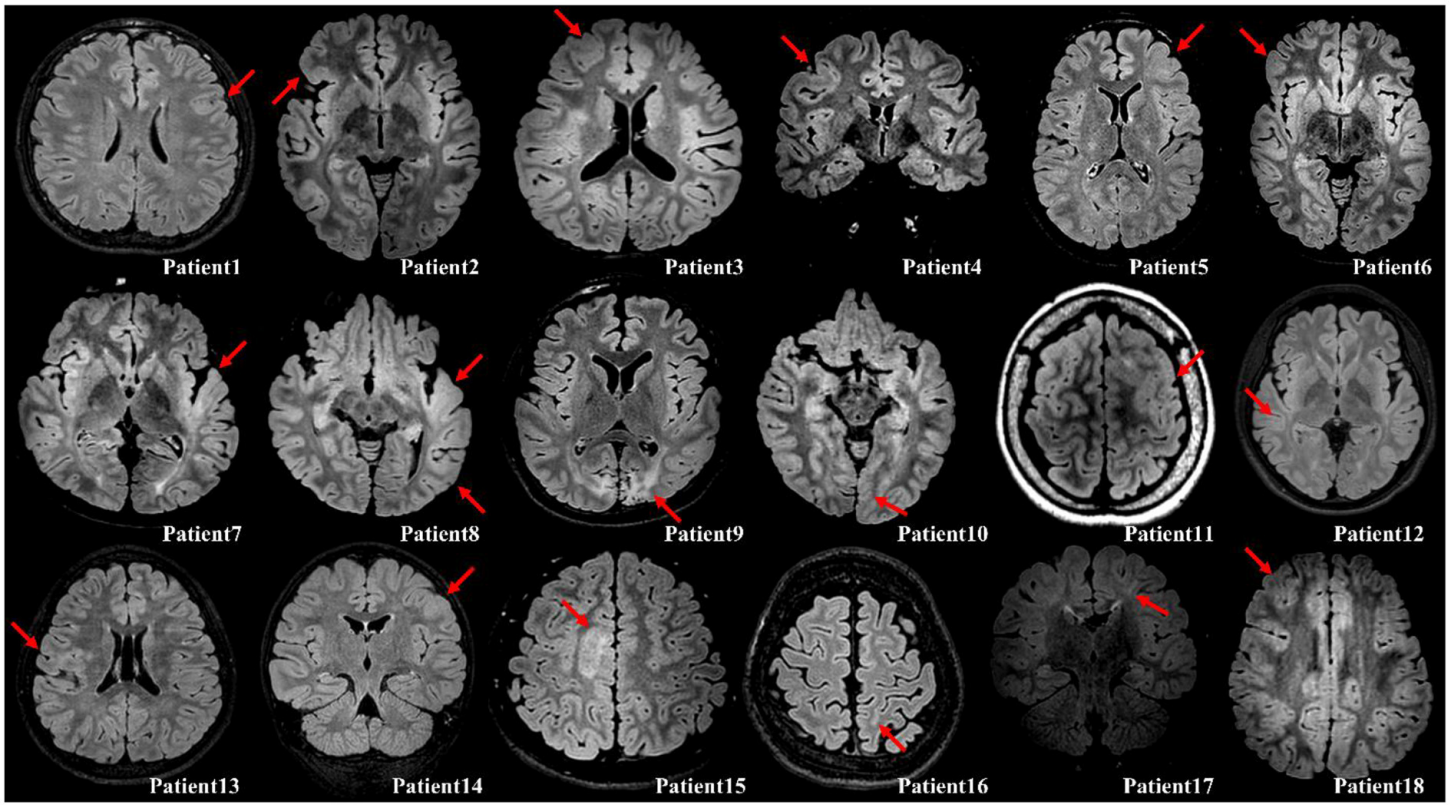
Illustration of the confirmed FCD on fluid attenuation inversion recovery images with either axial or coronal planes (red arrow). Patient 1, Cortical thickening; Patient 2, Cortical thickening and blurring of gray–white matter junction; Patient 3, Cortical thickening; Patient 4, Cortical thickening; Patient 5, Cortical thickening and blurring of gray–white matter junction; Patient 6, Cortical thickening; Patient 7, Cortical thickening, blurring of gray–white matter junction, and subcortical white matter hyperintensity; Patient 8, Cortical thickening and blurring of gray–white matter junction; Patient 9, Destruction on brain gray–white matter on both occipital (Lt>Rt); Patient 10, Cortical thickening and blurring of gray–white matter junction; Patient 11, Cortical thickening and blurring of gray–white matter junction; Patient 12, Cortical thickening and blurring of gray–white matter junction; Patient 13, Cortical thickening, blurring of gray–white matter junction, and subcortical white matter hyperintensity; Patient 14, Cortical thickening and blurring of gray–white matter junction; Patient 15, Cortical thickening and subcortical white matter hyperintensity; Patient 16, Blurring of gray–white matter junction; Patient 17, Subcortical white matter hyperintensity with transmantle sign; Patient 18, Cortical thickening and blurring of gray–white matter junction.

This retrospective study was approved by the Institutional Review Board of Yonsei University, College of Medicine, Seoul, South Korea. Eighteen patients were selected from our database (Supplementary Table 1), who had undergone resection surgery at Severance Children's Hospital from 2016 to 2020 under the following inclusion criteria: (1) patients who were diagnosed with focal epilepsy with FCD type II and were drug–resistant to at least two or three anti–seizure medications; (2) patients who had undergone grid and depth electrode insertion with video EEG recording; (3) patients who had good surgical outcomes with Engel class I or II; (4) patients who had undergone imaging studies such as MRI, computed tomography, and PET. A neuropathologist made the pathological diagnosis from resected brain specimen. FCD type II was diagnosed by the microscopic findings with cortical lamination disruption and the presence of dysmorphic neurons and/or balloon cells (15). All the eighteen patients have confirmed FCD on fluid attenuation inversion recovery images with the diagnosis demonstrated in Figure 1. Of these, eight patients were excluded owing to the following exclusion criteria: (1) patients with shorter ECoG recordings (5 days or less), which limited the selection of sufficient epochs for analysis; and (2) patients who had more than one resection surgery.

### ECoG data acquisition and processing

ECoG data were obtained using a combination of subdural grid electrodes (1 × 4, 1 × 8, 2 × 8, 3 × 8, 4 × 8, 2 × 5, and 4 × 5 matrices with 1 cm spacing between electrode contacts) and depth electrodes (8 channels with 1 cm spacing between electrode contacts) with a digital EEG acquisition system (Grass Telefactor, Astro–Med Inc, and Xltek NeuroWorks, Natus Medical Inc., Wisconsin, USA). Owing to individualized circumstances from presurgical evaluation to different system settings, the number of electrodes varied from 64 to 128, and the sampling rate ranged from 200 to 2,048 Hz. The average recording value of all electrodes was set as the reference because this method provides biased estimates of reference–independent potentials (16). Then, channels that contained artifacts such as electrical interference were removed, data were down–sampled to 200 Hz, a bandpass filter was used to provide cut–off frequencies between 0.5 and 66.7 Hz (high–cutoff filter 66.6 Hz was set to one–third sampling rate to avoid aliasing), and a notch filter was used to provide a cut–off frequency of 60 Hz.

### Time–frequency analysis of phase transfer entropy, graph theory analysis, and power spectrum compensation

#### Phase transfer entropy

Communication between neurons, which is presented by signal synchrony and is widely assumed to be non-linear, is the basic mechanism behind information processing inside the brain. Early studies have demonstrated that non-linear analysis can aid in the localization of EZ using IEDs (17), ictal activity (18), or interictal to ictal transition (19, 20). Furthermore, various studies have found non-linearity in large–scale brain networks in resting states, Parkinson's disease, and in patients with epilepsy (21). To evaluate the non-linear causality, transfer entropy was proposed by measuring the extent to which the lagged variable (additional information) reduces the uncertainty in the residuals of the model (22). More information can be found in the section of phase transfer entropy from the Supplementary material.

#### Analysis window selection for graph theory analysis and power spectrum compensation

**Figure 2:**
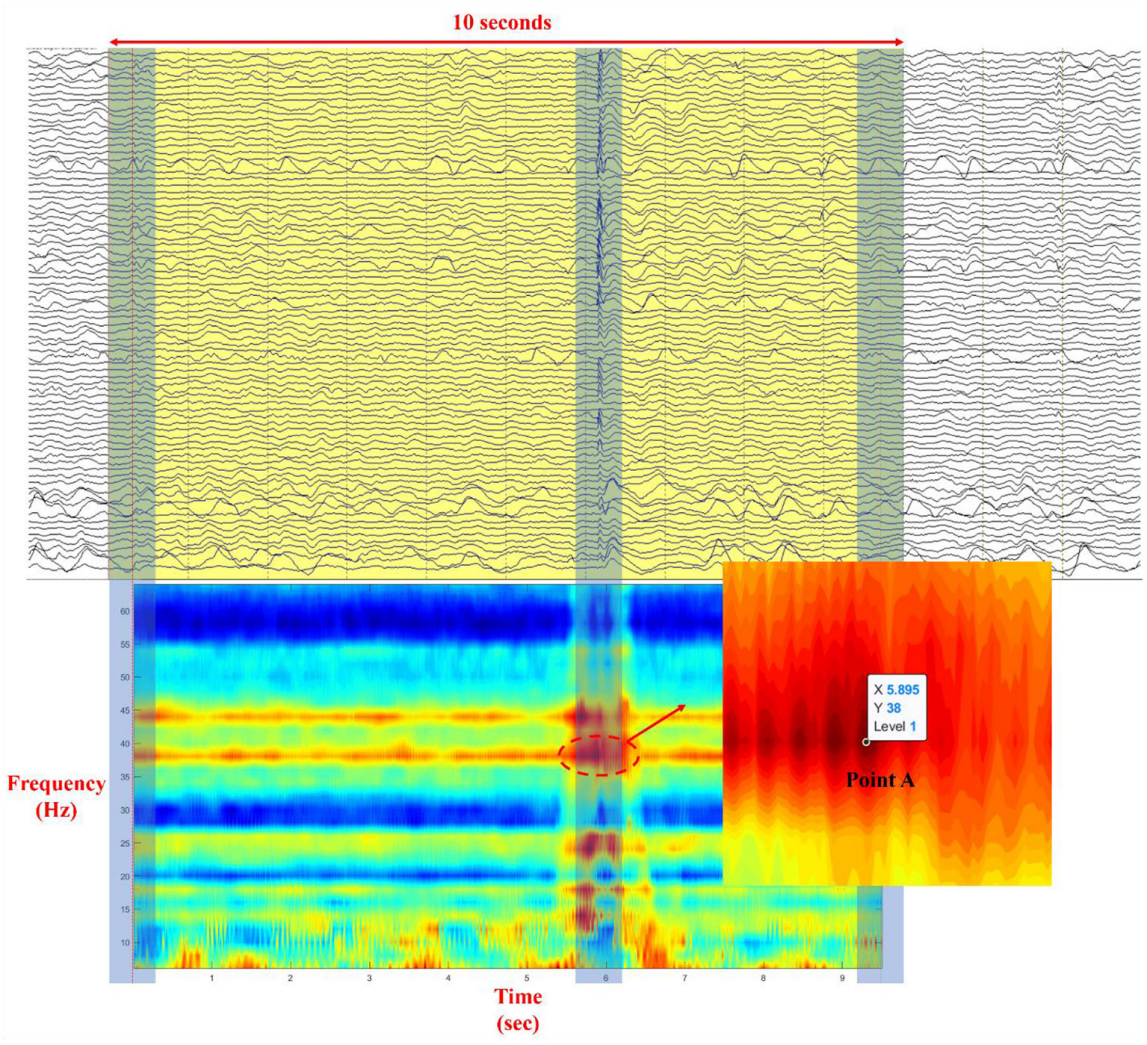
Illustration of the process of selection of point A in a time–frequency plot (Each time–frequency point in the plot represents the level of phase synchrony at a specific time point and a given frequency; ECoG data was illustrated with a bandpass filter to provide cut–off frequencies between 0.5 and 66.6 Hz and with a notch filter to provide cut–off frequency at 60 Hz).

The phase can easily be derived from a broadband signal using the Hilbert transform or wavelet convolution. However, to obtain a clear physical meaning of the phase and power spectrum, filtration should be used to extract the narrow–band frequency from the background brain activity (23). Time–frequency analysis of the phase transfer entropy was performed to find a point that has the highest value of phase transfer entropy with a specific frequency and time point. For each patient, 100 epochs (IEDs) in ECoG were strictly selected (IEDs pattern for each patient has a similar duration of discharges) for the time–frequency analysis of phase transfer entropy with a frequency range of 3–65 Hz and a step of 2 Hz. A FIR filter was used to prevent the phase distortion. As Figure 2 illustrates, a time–frequency plot was calculated from a 10–sec long epoch using a sliding window with a width of 500–msec, and the phase transfer entropy reached the highest level at point A (enlarged picture; maximum = 1) during the IEDs, where point A is located at a specific time point and a given frequency. The extracted time and frequency information was used to build an adjacency matrix from a 500 ms analysis window, where the selected point A was located in the center of the analysis window, and a 70% threshold was applied to the matrix, as this value provides a good balance for calculating betweenness centrality (BC), node out–degree (OD), and node in–degree (ID) based on our analysis experience. For more information about the results of different thresholds, refer to Supplementary Table 3 and Supplementary Figures 2–11. Simultaneously, the maximum power spectral values of each electrode were extracted from the analysis window for the latter power spectrum compensation. Later, graph theory analysis (13) was used to calculate seven parameters of each electrode: BC, clustering coefficient (CC), local efficiency (LE), OD, node out–strength (OS), ID, and node in–strength (IS), which were further averaged from one hundred epochs. Finally, the power spectrum compensation was introduced by multiplying the averaged power spectral value with all seven averaged parameters, one by one, and electrode by electrode.

### Information on electrodes and statistical analysis

The information on the ECoG location and the number of electrodes in the EZ, MZ, and NZ can be seen in Supplementary Figure 1 and Supplementary Table 2. Within each zone, the values of each electrode are listed in the column, and the Mann–Whitney *U*-test (*p* < 0.05; two–tailed) was applied to the statistical analysis between EZ, MZ, and NZ. Additionally, logistic regression was used for the probability calculation of EZ vs. MZ and EZ vs. NZ using all brain parameters. A cut–off probability for classification was set as 0.5: above 0.5 is classified as EZ and below 0.5 is classified as MZ or NZ.

## Results

Five patients each with frontal and extra–frontal FCD were evaluated. The IED types processed in this cohort can be found in Supplementary Table 1, where polyspike–wave complexes were used for patients no. 1 and no. 4, and sharp–wave discharges were used for the rest of patients. The two–tailed Mann–Whitney *U*-test (*p* < 0.05) was applied to evaluate EZ–MZ, EZ–NZ, and MZ–NZ for each parameter, as presented in Table 1. We observed that the parameters CC, LE, OD, and OS had the most significant differences among all ten patients. According to the Supplementary Table 4, and Supplementary Figures 12, 13, predictive accuracy from logistic regression is 65.44 ± 36.93% for EZ and 74.35 ± 29.77% for MZ from EZ vs. MZ, and 73.19 ± 10.45% for EZ and 92.86 ± 4.24% for NZ from EZ vs. NZ. Seven parameters were divided into three groups for a detailed evaluation with the inclusion of relevant figures according to their definitions in subsections Local segregation, Direct interaction, and Centrality. In this study, 100 epochs (IEDs) for each patient were strictly selected and used for analysis.

**Table 1:** Mann–Whitney *U*-test (two–tailed) for comparative analysis between EZ, MZ, and NZ for each parameter.

***p*< 0.05**		**CC**	**LE**	**OD**	**OS**	**ID**	**IS**	**BC**
P1	EZ–MZ	0.16	0.16	0.04^⋆^	0.04^⋆^	0.58	0.64	0.16
	EZ–NZ	<0.001^⋆⋆^	<0.001^⋆⋆^	<0.001^⋆⋆^	<0.001^⋆⋆^	0.54	0.54	0.89
	MZ–NZ	<0.001^⋆⋆⋆^	<0.001^⋆⋆⋆^	<0.001^⋆⋆⋆^	<0.001^⋆⋆⋆^	<0.001^##^	0.006^#^	0.04^#^
P2	EZ–MZ	0.003^⋆^	<0.001^⋆⋆^	<0.001^⋆⋆^	<0.001^⋆⋆^	0.13	0.13	0.01^⋆^
	EZ–NZ	<0.001^⋆⋆^	<0.001^⋆⋆^	<0.001^⋆⋆^	<0.001^⋆⋆^	0.03^⋆^	0.03^⋆^	0.006^⋆^
	MZ–NZ	0.008^⋆^	0.007^⋆^	0.005^⋆^	0.006^⋆^	0.12	0.13	0.13
P3	EZ–MZ	<0.001^⋆⋆^	<0.001^⋆⋆^	<0.001^⋆⋆^	<0.001^⋆⋆^	0.008^⋆^	0.008^⋆^	0.26
	EZ–NZ	0.003^⋆^	0.003^⋆^	0.007^⋆^	0.007^⋆^	<0.001^⋆⋆^	<0.001^⋆⋆^	0.91
	MZ–NZ	0.11	0.11	0.04^#^	0.03^#^	0.71	0.66	0.26
P4	EZ–MZ	0.002^⋆^	0.002^⋆^	0.003^⋆^	0.003^⋆^	0.02^#^	0.022^#^	0.99
	EZ–NZ	<0.001^⋆⋆^	<0.001^⋆⋆^	<0.001^⋆⋆^	<0.001^⋆⋆^	0.003^#^	0.003^#^	0.34
	MZ–NZ	0.001^⋆^	0.001^⋆^	0.002^⋆^	0.002^⋆^	0.90	0.79	0.09
P5	EZ–MZ	<0.001^⋆⋆⋆^	<0.001^⋆⋆⋆^	<0.001^⋆⋆⋆^	<0.001^⋆⋆⋆^	0.28	0.26	0.04^#^
	EZ–NZ	<0.001^⋆⋆⋆^	<0.001^⋆⋆⋆^	<0.001^⋆⋆⋆^	<0.001^⋆⋆⋆^	0.23	0.30	0.003^#^
	MZ–NZ	0.03^⋆^	0.03^⋆^	0.04^⋆^	0.04^⋆^	0.44	0.50	0.25
P6	EZ–MZ	0.01^⋆^	0.01^⋆^	0.02^⋆^	0.02^⋆^	0.16	0.17	0.34
	EZ–NZ	<0.001^⋆⋆⋆^	<0.001^⋆⋆⋆^	<0.001^⋆⋆⋆^	<0.001^⋆⋆⋆^	<0.001^⋆⋆⋆^	<0.001^⋆⋆⋆^	0.82
	MZ–NZ	0.45	0.47	0.46	0.43	0.04^⋆^	0.05	0.31
P7	EZ–MZ	<0.001^⋆⋆^	<0.001^⋆⋆^	<0.001^⋆⋆^	<0.001^⋆⋆^	0.04^⋆^	0.05	0.79
	EZ–NZ	<0.001^⋆⋆^	<0.001^⋆⋆^	<0.001^⋆⋆^	<0.001^⋆⋆^	0.27	0.27	0.42
	MZ–NZ	0.75	0.75	0.70	0.67	0.49	0.47	0.70
P8	EZ–MZ	<0.001^⋆⋆^	<0.001^⋆⋆^	<0.001^⋆⋆^	<0.001^⋆⋆^	0.22	0.33	0.18
	EZ–NZ	<0.001^⋆⋆⋆^	<0.001^⋆⋆⋆^	<0.001^⋆⋆⋆^	<0.001^⋆⋆⋆^	0.08	0.13	<0.001^##^
	MZ–NZ	0.75	0.77	0.84	0.82	0.99	0.96	0.17
P9	EZ–MZ	0.01^⋆^	0.01^⋆^	0.01^⋆^	0.01^⋆^	0.04^⋆^	0.04^⋆^	0.98
	EZ–NZ	<0.001^⋆⋆^	<0.001^⋆⋆^	<0.001^⋆⋆^	<0.001^⋆⋆^	0.02^⋆^	0.02^⋆^	0.40
	MZ–NZ	0.21	0.22	0.24	0.24	0.28	0.28	0.35
P10	EZ–MZ	<0.001^⋆⋆^	<0.001^⋆⋆^	0.001^⋆^	0.001^⋆^	0.003^⋆^	0.004^⋆^	0.82
	EZ–NZ	<0.001^⋆⋆⋆^	<0.001^⋆⋆⋆^	<0.001^⋆⋆⋆^	<0.001^⋆⋆⋆^	<0.001^⋆⋆⋆^	<0.001^⋆⋆⋆^	0.23
	MZ–NZ	0.38	0.41	0.97	0.92	0.21	0.22	0.22

P1, patient 1; EZ, epileptogenic zone; MZ, margin zone; NZ, normal zone; BC, betweenness centrality; CC, clustering coefficient; LE, local efficiency; OD, out–degree; OS, out–strength; ID, in–degree; IS, in–strength;

⋆ represents p < 0.05 with EZ > MZ, EZ > NZ, or MZ > NZ;

⋆⋆ represents p < 0.001 with EZ > MZ, EZ > NZ, or MZ > NZ;

⋆⋆⋆ represents p < 0.00001 with EZ > MZ, EZ > NZ, or MZ > NZ;

# represents p < 0.05 with EZ < MZ, EZ < NZ, or MZ < NZ;

## represents p < 0.001 with EZ < MZ, EZ < NZ, or MZ < NZ.

### Local segregation

**Figure 3:**
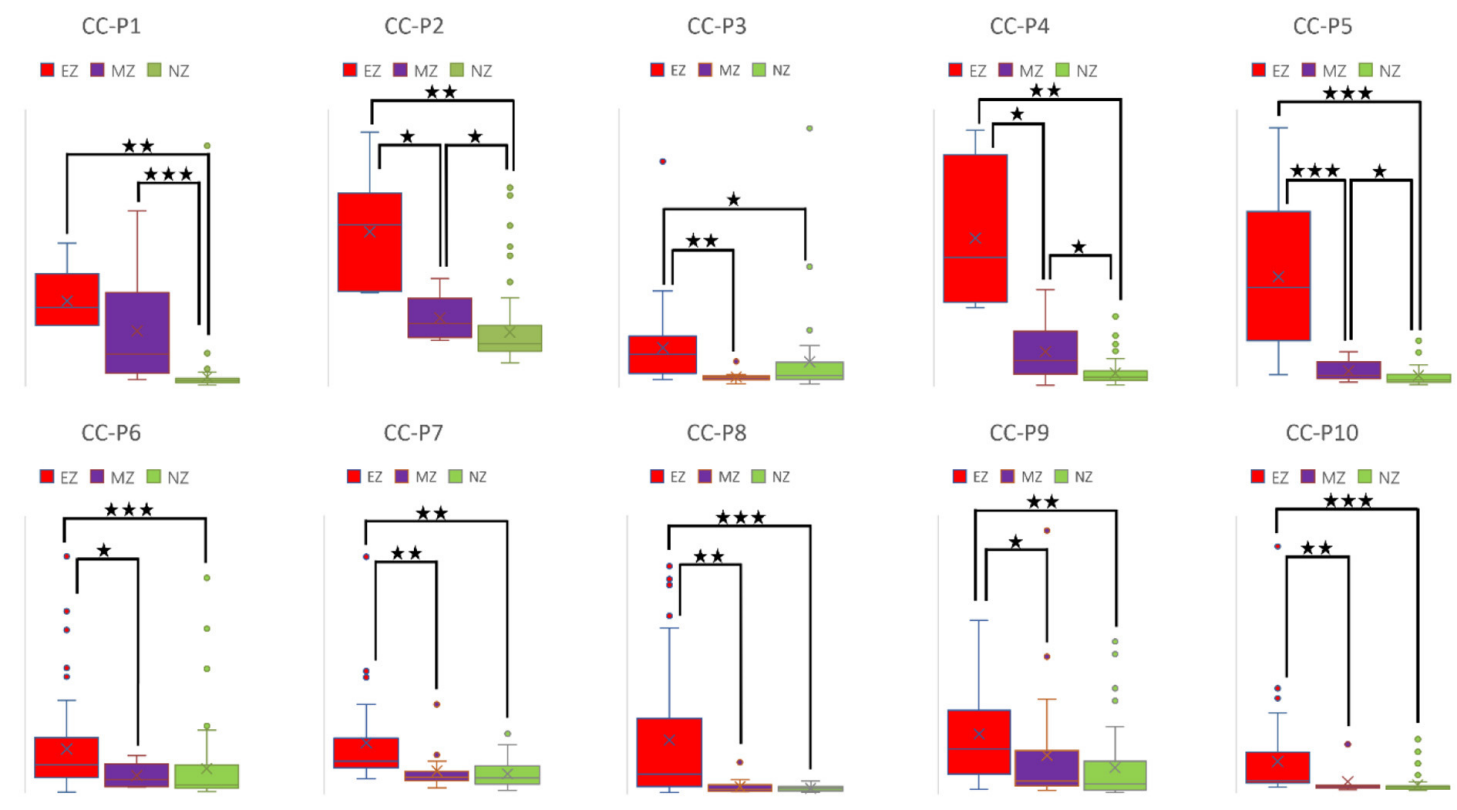
Box plot of CC of all ten patients for EZ, MZ, and NZ (P1 represents patient 1; ⋆ represents *p* < 0.05 with EZ > MZ, EZ > NZ, or MZ > NZ; ⋆⋆ represents *p* < 0.001 with EZ > MZ, EZ > NZ, or MZ > NZ; ⋆⋆⋆ represents *p* < 0.00001 with EZ > MZ, EZ > NZ, or MZ > NZ; Mann–Whitney *U*-test [two–tailed]). CC, clustering coefficient; EZ, epileptogenic zone; MZ, margin zone; NZ, normal zone.

**Figure 4:**
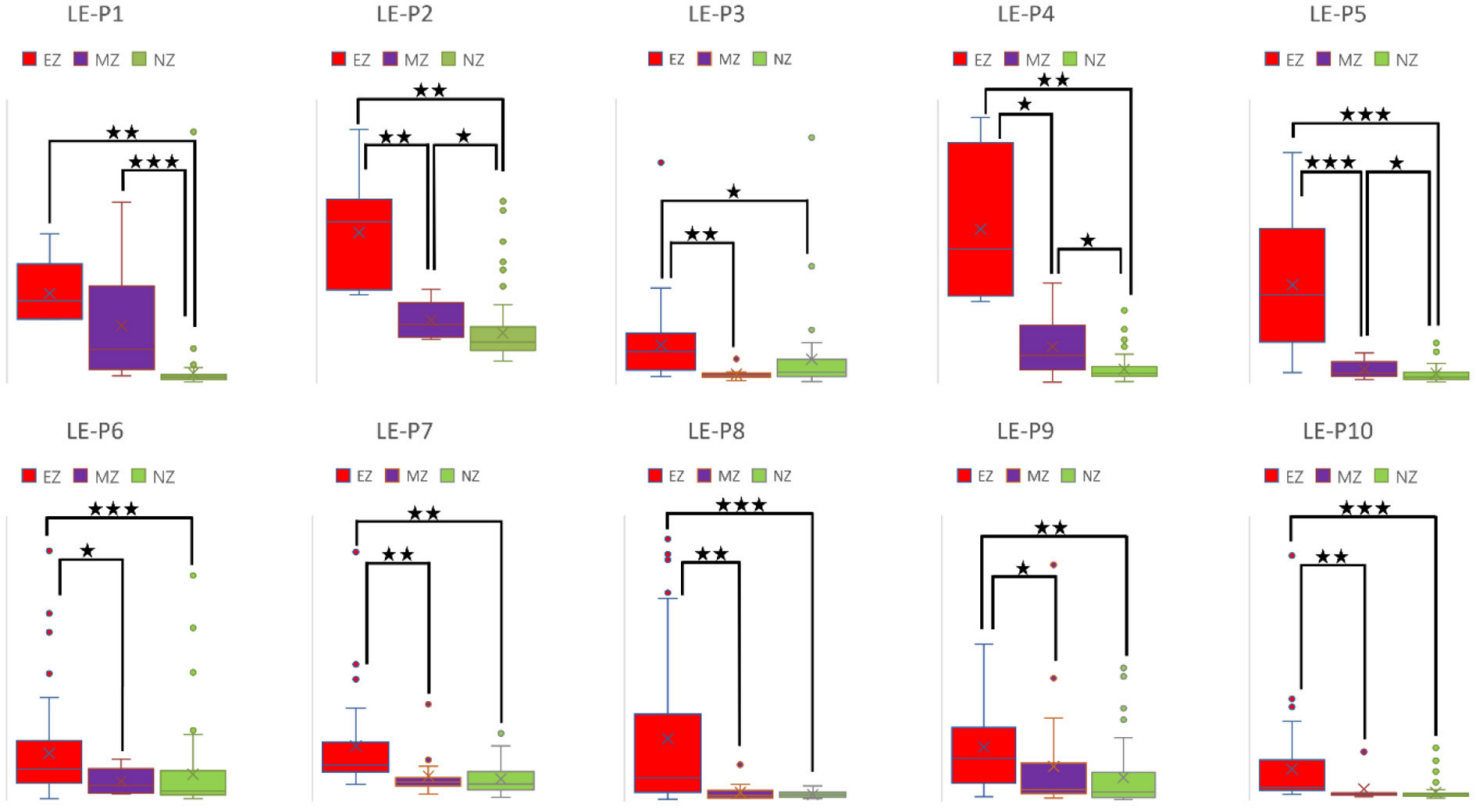
Box plot of LE of all ten patients for EZ, MZ, and NZ (P1 represents patient 1; ⋆ represents *p* < 0.05 with EZ > MZ, EZ > NZ, or MZ > NZ; ⋆⋆ represents *p* < 0.001 with EZ > MZ, EZ > NZ, or MZ > NZ; ⋆⋆⋆ represents *p* < 0.00001 with EZ > MZ, EZ > NZ, or MZ > NZ; Mann–Whitney *U*-test [two–tailed]). LE, local efficiency; EZ, epileptogenic zone; MZ, margin zone; NZ, normal zone.

CC and LE are common parameters for local segregation (24). CC measures the connection density between a node and its neighborhood. A node with a high CC value represents a strong connection with its neighbors, and together they form a cluster. LE measures the average efficiency of a node within its neighbors; thus, it is related to CC. In Figures 3, 4, for evaluating EZ–MZ and EZ–NZ, CC and LE were stable among all patients (EZ > MZ and EZ > NZ) except for patient 1 in EZ–MZ for both CC and LE (*p* = 0.16). For evaluating MZ–NZ, results were divided into two groups according to the location of FCD (frontal FCD: patients no. 1–5; extra frontal FCD: patients no. 6–10): patients no. 1, 2, 4, and 5 had significantly higher values of MZ (MZ > NZ) for both CC and LE, except for patient no. 3 in CC and LE (*p* = 0.11), while patients 6–10 who had extra–frontal FCD did not show any significant difference, which may imply an underlying anatomical cause.

### Direct interaction

**Figure 5:**
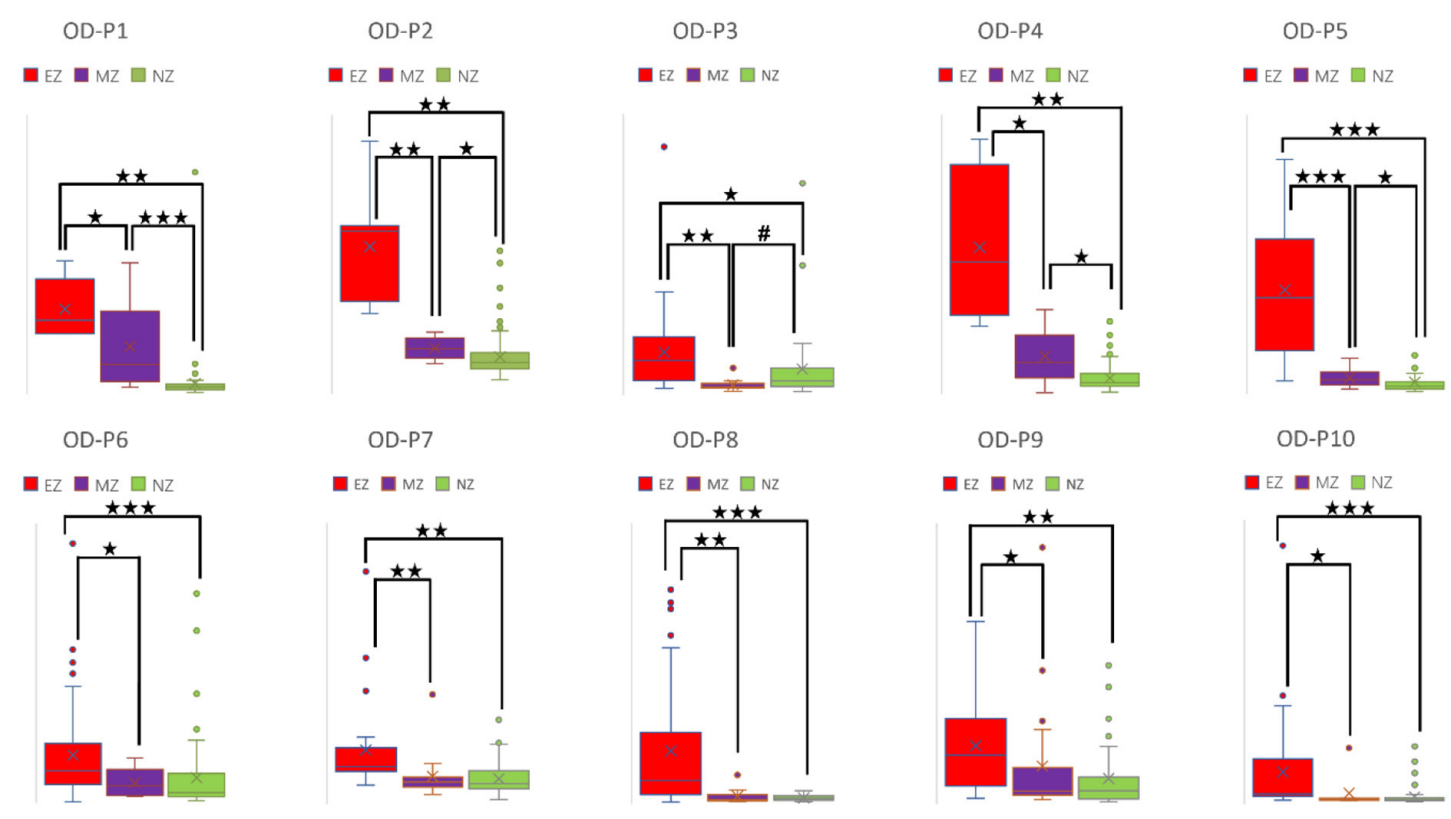
Box plot of OD of all ten patients for EZ, MZ, and NZ (P1 represents patient 1; ⋆ represents *p* < 0.05 with EZ > MZ, EZ > NZ, or MZ > NZ; ⋆⋆ represents *p* < 0.001 with EZ > MZ, EZ > NZ, or MZ > NZ; ⋆⋆⋆ represents *p* < 0.00001 with EZ > MZ, EZ > NZ, or MZ > NZ; ^#^ represents *p* < 0.05 with EZ < MZ, EZ < NZ, or MZ < NZ; Mann-Whitney *U* test [two–tailed]). OD, out–degree; EZ, epileptogenic zone; MZ, margin zone; NZ, normal zone.

**Figure 6:**
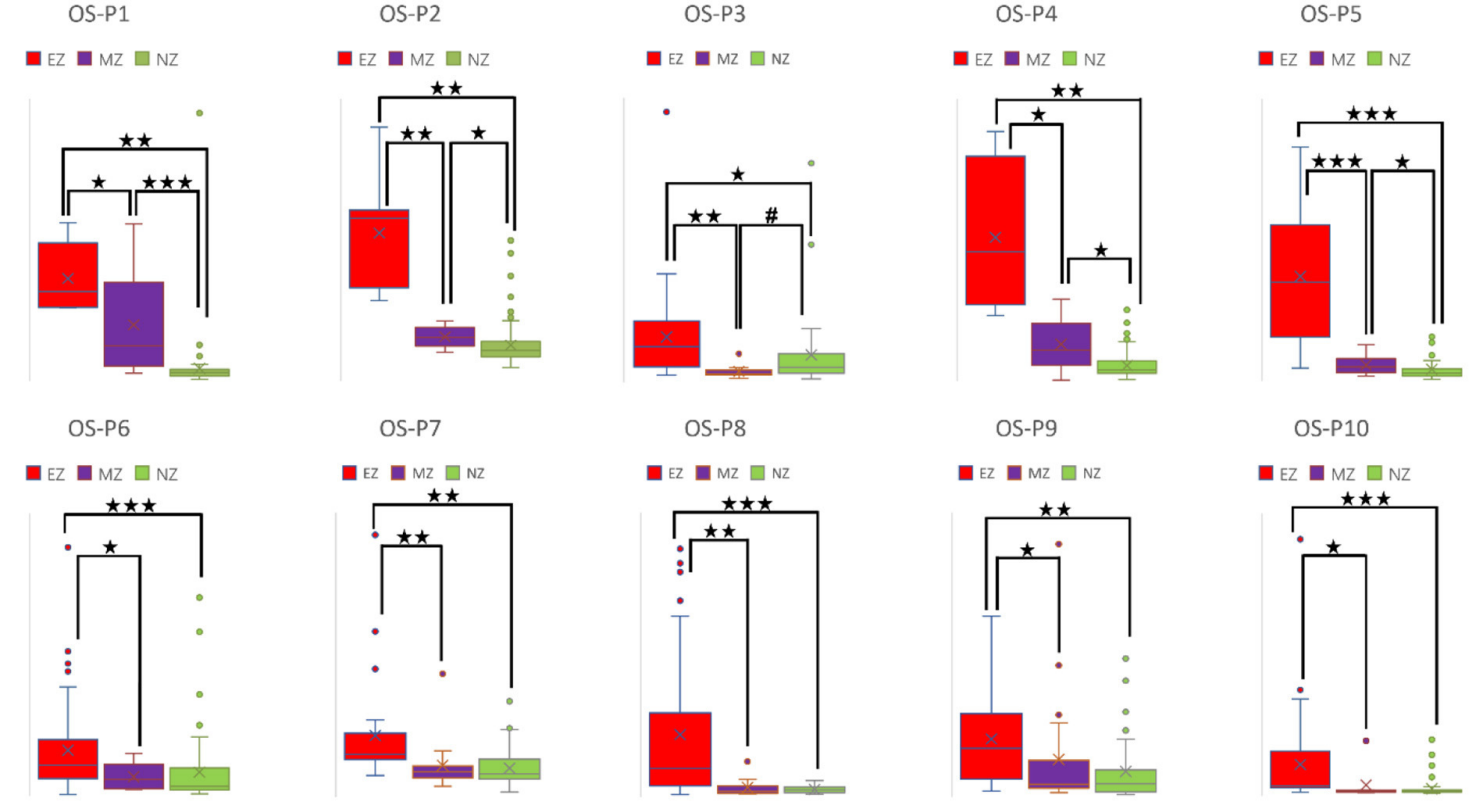
Box plot of OS of all ten patients for EZ, MZ, and NZ (P1 represents patient 1; ⋆ represents *p* < 0.05 with EZ > MZ, EZ > NZ, or MZ > NZ; ⋆⋆ represents *p* < 0.001 with EZ > MZ, EZ > NZ, or MZ > NZ; ⋆⋆⋆ represents *p* < 0.00001 with EZ > MZ, EZ > NZ, or MZ > NZ; ^#^ represents *p* < 0.05 with EZ < MZ, EZ < NZ, or MZ < NZ; Mann-Whitney *U* test [two–tailed]). OS, out–strength; EZ, epileptogenic zone; MZ, margin zone; NZ, normal zone.

For a directed connectivity network, OD/ID represents the number of outward/inward edges of a node. Similarly, OS/IS represents the summation of the weights of the outward/inward edges of a node. As demonstrated in Figures 5, 6, all patients had EZ > MZ and EZ > NZ for OD and OS. For evaluating MZ–NZ, results were divided into two groups according to the location of FCD (frontal FCD: patients no. 1–5; extra frontal FCD: patients no. 6–10): Patients no. 1, 2, 4, 5 showed MZ > NZ for both OD and OS, whereas patients no. 6–10 did not exhibit this finding. According to Table 1, parameters of ID and IS showed fewer significant differences, and the relationships among these zones were complicated. ID showed EZ > MZ in patients no. 3, 7, 9, and 10; EZ > NZ in patients no. 2, 3, 6, 9, and 10; MZ > NZ in patient no. 6; EZ < MZ and EZ < NZ in patient no. 4; and MZ < NZ in patient no. 1. Patients no. 5 and 8 had no significant differences in any comparison group. IS showed EZ > MZ in patients no. 3, 9, and 10; EZ > NZ in patients no. 2, 3, 6, 9, and 10; EZ < MZ and EZ < NZ in patient no. 4; and MZ < NZ in patient no. 1. Patients no. 5, 7, and 8 did not demonstrate any significant difference in any comparison group.

### Centrality

The BC of a selected node is defined as the fraction of all shortest paths in the network that a particular node participates in. A network with a lower short path length means that each node can be reached from any other node with fewer edges. The destruction of a node with a high BC value would significantly impact the network because it is at the intersection of many short paths (24). The results of BC are presented in Table 1, where we observe EZ > MZ and EZ > NZ in patient no. 2, EZ < MZ in patient no. 5, EZ < NZ in patient no. 5 and 8, and MZ < NZ in patient no. 1. Patients no. 3, 4, 6, 7, 9, and 10 did not demonstrate any significant difference in all the comparison groups.

## Discussion

Here, we demonstrated that brain network analysis—based on the combination of time–frequency analysis of phase transfer entropy, graph theory analysis, and power spectrum compensation—could aid in the identification of EZ. Furthermore, we proved that four brain network parameters, CC, LE, OD, and OS, could be potential biomarkers for differentiating EZ from MZ and NZ (EZ > MZ and EZ > NZ).

### Methodology of brain network analysis based on IEDs

Frequency, power spectrum, and phase are the three components of neuronal oscillation (25). Different frequency bands are responsible for specific roles, as neuronal oscillation generates synchrony across distinct brain regions to realize different cognitive functions (26) or to support epileptiform activity (27). A previous study reported that higher spectral power of a periodic discharge pattern was associated with a higher risk of seizures in time–frequency analysis, emphasizing the importance of the power spectrum as an indicator of epileptogenesis (28). However, the frequency or power spectrum cannot reflect the coordination behind neurons across different brain regions (25). Contrarily, the phase, which shows the position of a signal at a specific time point within a given oscillation cycle, plays a crucial role in coordinating the communication between anatomically distributed brain regions (25). Instead of using cross–frequency coupling, we extracted the phase and power spectrum in the same frequency band for analysis.

Although the morphology of a generalized seizure visually represents a highly synchronized pattern, analysis reveals that synchrony during this ictal activity is imperfect (29). Ortega et al. found that phase synchronization and linear correlation performed better in the analysis of synchronization clusters from interictal activity in patients with temporal lobe epilepsy. Receiver operating characteristic analysis indicated that seizure control was achieved by removing the brain cortices that produce synchronized sharp clusters (30). Meesters et al. introduced a framework to model the network interactions behind the IEDs. Independent component analysis was performed to determine the interdependency of brain regions using synchronized spikes. They found that this approach could aid the visual review of ECoG and promises an increased success rate of resection surgery (31). Using time–frequency analysis of phase transfer entropy with a sliding window of 500 ms, we found that the phase synchrony value reached its peak at point A with a specific time point and a given frequency (Figure 2) during the IED period. Point A was used to select an analysis window that was fed for brain network analysis.

Epilepsy is a network disease of the brain; therefore, with graph theory analysis of functional and effective connectivity networks, it is possible to derive from the interaction and analyze the causal relationship between signals during different events. By selecting and analyzing IEDs during the awake state, Keller et al. found that IEDs in patients with epilepsy reflect a complex and dynamic network presentation, resulting from a heterogeneous population of synchronous neurons (32). Based on the analysis results of IEDs, Ortega et al. found that synchronization analysis could be used to functionally map patients with temporal lobe epilepsy, as the synchronous ECoG was highly different between specific brain regions and the temporal lobe (EZ) (30). Several brain parameters can be divided into local segregation, global integration, centrality, etc., and each parameter carries either a single value of a network, or the value of each node (24). We selected some parameters that have individual values for all nodes, and, we divided the electrodes into EZ, MZ, and NZ for comparison.

Spike–wave discharges, polyspikes, polyspike–wave complexes, beta/gamma oscillations, and HFOs are the common IEDs types. Cuello–Oderiz et al. defined the typical IEDs that show either spike or polyspike above 2 Hz or spike or polyspike interrupted by a flat period below 2 Hz (33). Hu et al. mentioned that the shape of IEDs could impact their results, and thus, they used spike–wave discharges since this type has the greatest potential to influence the calculation of brain connectivity (34). In our study, polyspike–wave complexes were used for patients no. 1 and no. 4, who had FCD in the primary functional cortices (motor and sensory) and sharp–wave discharges were used for the rest of patients, who had FCD with motor and sensory cortices spared. We think polyspike–wave complexes have correlated with the FCD location since this pattern both exits in the ictal (35) and interictal events (36) from the motor and sensory cortices. In our analysis, wavelet convolution was used to extract the information from narrow bands. Compared to the spike–wave discharges, the slow wave component from polyspike–wave complexes has more effects during the convolution process. According to the analysis results, we think both patterns are typical IEDs, which carry the information about underlying pathological activity and have the greatest potential to be used for analysis.

With the occurrence of digital EEG, data recorded with high sampling rate are preferred to support the pre–surgical evaluation in the identification of two biomarkers: < inlinelist> (1) low voltage fast activity at the start of ictal events; (2) HFOs during interictal events < /inlinelist> (37). Since our patients' ECoG data have different sampling rates (200 Hz to 2,048 Hz), we down–sampled the data with a higher sampling rate and kept the sampling frequency of 200 Hz among all patients to make sure the 500 msec analysis window will contain the same time point for the later analysis. According to the Nyquist sampling theorem, a continuous–time signal can be perfectly reconstructed from its sampling points, given that the waveform is sampled at least twice as quickly as its highest frequency component (38). Additionally, a high–cutoff filter 66.6 Hz was set to one–third sampling rate (200 Hz) to avoid aliasing. In our analysis, rather than beta/gamma oscillations or HFOs, polyspike–wave complexes and spike–wave discharges are used, and both can be sufficiently reconstructed and analyzed with a sampling frequency rate of 200 Hz.

### Brain network parameters as biomarkers for differentiating EZ

A high CC value was observed in patients with mesial temporal lobe epilepsy using sEEG during the interictal period (39). In a comparison study that used electrical source imaging (ESI) and directed functional network from low–density EEG, ESI, summed outflow, and efficiency were concordant in 76% of patients within the presumed EZ from the interictal spike (40). In our study, CC and LE were stable among all patients for evaluation of EZ–MZ and EZ–NZ (EZ > MZ and EZ > NZ), except for patient no. 1 in EZ–MZ (*p* = 0.16 for both CC and LE). In a stereo–EEG study of epilepsy patients with FCD type II, significantly higher OD values in the gamma band helped differentiate the EZ from other brain regions during interictal, preictal, and ictal events (41). Higher total and OS values from the resected brain tissue were found in the gamma and ripple bands in patients with a good outcome (42). We found that OD and OS were the best parameters for differentiating EZ from MZ and NZ, with all patients demonstrating significant differences in the comparison groups; ID and IS did not demonstrate this feature. Electrodes with a high BC value are hubs, which may play an important role in inhibiting or terminating seizures during interictal and post–ictal states, and resection of these nodes is not necessary to achieve seizure freedom (43). According to our results, BC was the worst parameter to differentiate EZ from MZ and NZ, with most patients demonstrating no significant differences in all comparison groups. In a stereotactic–EEG study on the EZ, propagation zone (PZ), and non–involved zone, Lagarde et al. found that functional connectivity is stronger within the EZ and PZ during the interictal event, indicating a reinforced network within epileptic cortices (EZ and PZ) with a gradual organization (44). We found that there is a significant difference for EZ–MZ (EZ > MZ) and EZ–NZ (EZ > NZ) in CC, LE, OD, and OD (except for patient no. 1 for EZ–MZ in CC and LE). According to Figures 3–6 that show higher CC and LE values in the EZ, representing strong interconnections with its neighbors; and higher OD and OS values in the EZ, indicating dense and strong connections pointing outside the EZ. This may imply that during the IED period, the epileptogenic brain cortex is more likely to build a strong and dense connection not only with its neighbors, which have a direct and close relationship with the EZ, but also between distant regions, which have network connections with the EZ.

### Limitations

Several experimental parameters influence the analysis and should be tuned and used with caution. The threshold applied to the adjacency matrix affects the results as we tried 30, 50, and 70%, and found 70% worked the best. Although this value may not be optimal, further exploration is required. The width of the sliding window is another influential parameter. 250 msec and 1 sec is not suggested since the former was unstable during the calculation due to the limited window length and the latter would introduce bias to the power spectrum compensation if IEDs have after–going slow waves containing high amplitude. Owing to the inadequate knowledge about the role of IEDs in human epilepsy, questions regarding IEDs continue to persist, for example, whether the underlying cellular and network mechanisms are different, regardless of the similar morphology of IEDs in EEG (45). IEDs types used for analysis are polyspike–wave complexes for patients no. 1 and no. 4, and spike–wave discharges for the rest of patients. Analysis results based on the other IEDs types, especially those for the high frequency activity (beta/gamma oscillations and HFOs) require future exploration with data recorded using a high sampling rate (19, 20). From the 100 epochs of each patient, most frequencies extracted at point A (Figure 2) have a fixed value. Others have fluctuations within a reasonable range (±4 Hz). Patient selection bias is the most important limitation of this study, given that most patients had frontal FCD, and all the patients had good surgical outcomes (9 Engel class I and 1 Engel class II). Additionally, more centers are moving toward sEEG, which can target deep brain structures with anatomical accuracy from both hemispheres. Further exploration and analysis are required on this topic with more patients included from Engel class I to IV. We proved that four brain network parameters, CC, LE, OD, and OS, are useful for differentiating EZ from MZ and NZ (EZ > MZ and EZ > NZ). However, the differentiation of MZ and NZ is beyond the scope of the current work, and future studies are needed to further explore this difference.

## Conclusion

In this study, based on the analysis of IEDs, we combined frequency, power spectrum, and phase for investigating potential biomarkers for the identification of EZ in pediatric focal epilepsy patients with FCD type II by using time–frequency analysis of phase transfer entropy, graph theory analysis, and power spectrum compensation. The current information regarding IEDs is sometimes ambiguous, and its clinical significance (role of IEDs in seizure and epilepsy) is unclear. A more comprehensive and deeper understanding of the cellular and network mechanisms underlying IEDs is required; further research should focus on the development of more complete models and more effective methods for epilepsy treatment.

## Data availability statement

The original contributions presented in the study are included in the article/Supplementary materials, further inquiries can be directed to the corresponding author/s.

## Ethics statement

The studies involving human participants were reviewed and approved by the Institutional Review Board of Yonsei University, College of Medicine. Written informed consent from the patients/participants' legal guardian/next of kin was not required to participate in this study in accordance with the national legislation and the institutional requirements'.

## Author contributions

ZW, YH, and HK designed the research. ZW and BN analyzed the data and drafted the manuscript. DY and BN selected the patients. All authors edited the manuscript, contributed to the article, and approved the submitted version of the manuscript.

## Funding

This research was funded by the National Research Foundation of Korea (Grant NRF−2020R1A2C1010803) that is sponsored by the Government of South Korea.

## Conflict of interest

The authors declare that the research was conducted in the absence of any commercial or financial relationships that could be construed as a potential conflict of interest.

## Publisher's note

All claims expressed in this article are solely those of the authors and do not necessarily represent those of their affiliated organizations, or those of the publisher, the editors and the reviewers. Any product that may be evaluated in this article, or claim that may be made by its manufacturer, is not guaranteed or endorsed by the publisher.

## References

[1] Gnatkovsky V De Curtis M Pastori C Cardinale F Lo Russo G Mai R. Biomarkers of epileptogenic zone defined by quantified stereo-EEG analysis. Epilepsia. (2014) 55:296–305. 10.1111/epi.12507 24417731

[2] Rasmussen T. Characteristics of a pure culture of frontal lobe epilepsy. Epilepsia. (1983) 24:482–93. 10.1111/j.1528-1157.1983.tb04919.x 6873005

[3] Khambhati AN Bassett DS Oommen BS Chen SH Lucas TH Davis KA. Recurring functional interactions predict network architecture of interictal and ictal states in neocortical epilepsy. Eneuro. (2017) 4:17. 10.1523/ENEURO.0091-16.2017 28303256 PMC5343278

[4] Carboni M Rubega M Iannotti GR De Stefano P Toscano G Tourbier S. The network integration of epileptic activity in relation to surgical outcome. Clinic Neurophysiol. (2019) 130:2193–202. 10.1016/j.clinph.2019.09.006 31669753

[5] Jobst BC Bartolomei F Diehl B Frauscher B Kahane P Minotti L. Intracranial EEG in the 21st Century. Epilepsy Curr. (2020) 20:180–8. 10.1177/1535759720934852 32677484 PMC7427159

[6] Jacobs J Wu JY Perucca P Zelmann R Mader M Dubeau F. Removing high-frequency oscillations a prospective multicenter study on seizure outcome. Neurology. (2018) 91:E1040–52. 10.1212/WNL.0000000000006158 30120133 PMC6140372

[7] Roehri N Pizzo F Mcgonigal A Bartolomei F Benar CG. Reply to “are spikes non-inferior to high-frequency oscillations?”. Annal Neurol. (2018) 83:870–71. 10.1002/ana.25200 29518267

[8] Thomschewski A Hincapie AS Frauscher B. Localization of the epileptogenic zone using high frequency oscillations. Front Neurol. (2019) 10:94. 10.3389/fneur.2019.00094 30804887 PMC6378911

[9] Engel JJr Bragin A Staba R. Nonictal EEG biomarkers for diagnosis and treatment. Epilepsia Open. (2018) 3:120–6. 10.1002/epi4.12233 30564770 PMC6293068

[10] Panzica F Varotto G Rotondi F Spreafico R Franceschetti S. Identification of the epileptogenic zone from stereo-eeg signals: a connectivity-graph theory approach. Front Neurol. (2013) 4:175. 10.3389/fneur.2013.00175 24223569 PMC3818576

[11] Shah P Bernabei JM Kini LG Ashourvan A Boccanfuso J Archer R. High interictal connectivity within the resection zone is associated with favorable post-surgical outcomes in focal epilepsy patients. Neuroimage-Clinic. (2019) 23:908. 10.1016/j.nicl.2019.101908 31491812 PMC6617333

[12] Gunnarsdottir K. M. Li A. Smith R. J. Kang J.-Y. Korzeniewska A. Crone N. E.. (2021). Source-sink connectivity: a novel interictal EEG marker for seizure localization. bioRxiv 2021:464594. 10.1101/2021.10.15.464594 PMC10200292 36412516

[13] Bullmore E Sporns O. Complex brain networks: graph theoretical analysis of structural and functional systems. Nat Rev Neurosci. (2009) 10:186. 10.1038/nrn2575 19190637

[14] Wang ZJ Kim ES Noh BH Liang JG Lee D Hur YJ. Alteration in brain connectivity in patients with Dravet syndrome after vagus nerve stimulation (VNS): exploration of its effectiveness using graph theory analysis with electroencephalography. J Neural Eng. (2020) 17:45. 10.1088/1741-2552/ab914f 32380482

[15] Kwon HE Eom S Kang HC Lee JS Kim SH Kim DS. Surgical treatment of pediatric focal cortical dysplasia clinical spectrum and surgical outcome. Neurology. (2016) 87:945–51. 10.1212/WNL.0000000000003042 27466475

[16] Nunez PL Srinivasan R. Electric Fields of the Brain: The Neurophysics of EEG. Oxford: Oxford University Press (2006).

[17] Le Van Quyen M Martinerie J Adam C Varela FJ. Non-linear analyses of interictal EEG map the brain interdependences in human focal epilepsy. Physica D-Non-linear Phenomena. (1999) 127:250–66. 10.1016/S0167-2789(98)00258-9

[18] Martinerie J Adam C Le Van Quyen M Baulac M Clemenceau S Renault B. Epileptic seizures can be anticipated by non-linear analysis. Nat Med. (1998) 4:1173–6. 10.1038/2667 9771751

[19] Grinenko O Li J Mosher JC Wang IZ Bulacio JC Gonzalez-Martinez J. A fingerprint of the epileptogenic zone in human epilepsies. Brain. (2018) 141:117–31. 10.1093/brain/awx306 29253102 PMC5837527

[20] Li J Grinenko O Mosher JC Gonzalez-Martinez J Leahy RM Chauvel P. Learning to define an electrical biomarker of the epileptogenic zone. Hum Brain Mapp. (2020) 41:429–41. 10.1002/hbm.24813 31609058 PMC7268034

[21] Ferdousi M Babaie-Janvier T Robinson PA. Non-linear wave-wave interactions in the brain. J Theor Biol. (2020) 500:110308. 10.1016/j.jtbi.2020.110308 32389568

[22] Schreiber T. Measuring information transfer. Phys Rev Lett. (2000) 85:461–4. 10.1103/PhysRevLett.85.461 10991308

[23] Le Van Quyen M Foucher J Lachaux JP Rodriguez E Lutz A Martinerie J. Comparison of Hilbert transform and wavelet methods for the analysis of neuronal synchrony. J Neurosci Methods. (2001) 111:83–98. 10.1016/S0165-0270(01)00372-7 11595276

[24] Sporns O. Networks of the Brain. Cambridge, Mass.: MIT Press (2011).

[25] Lobier M Siebenhuhner F Palva S Palva JM. Phase transfer entropy: a novel phase-based measure for directed connectivity in networks coupled by oscillatory interactions. Neuroimage. (2014) 85:853–72. 10.1016/j.neuroimage.2013.08.056 24007803

[26] Munia TTK Aviyente S. Time-frequency based phase-amplitude coupling measure for neuronal oscillations. Sci Rep. (2019) 9:2. 10.1038/s41598-019-48870-2 31455811 PMC6711999

[27] Gajic D Djurovic Z Gligorijevic J Di Gennaro S Savic-Gajic I. Detection of epileptiform activity in EEG signals based on time-frequency and non-linear analysis. Front Comput Neurosci. (2015) 9:38. 10.3389/fncom.2015.00038 25852534 PMC4371704

[28] Chen JH Zhou XQ Jin LR Lu Q Sun HY Liu Q. Can spectral power be used as a candidate seizure marker of the periodic discharges pattern? Front Neurol. (2021) 12:669. 10.3389/fneur.2021.642669 34194380 PMC8236598

[29] Dominguez LG Wennberg RA Gaetz W Cheyne D Snead OC Velazquez JLP. Enhanced synchrony in epileptiform activity? - local versus distant phase synchronization in generalized seizures. J Neurosci. (2005) 25:8077–84. 10.1523/JNEUROSCI.1046-05.2005 16135765 PMC6725453

[30] Ortega GJ De La Prida LM Sola RG Pastor J. Synchronization clusters of interictal activity in the lateral temporal cortex of epileptic patients: Intraoperative electrocorticographic analysis. Epilepsia. (2008) 49:269–80. 10.1111/j.1528-1167.2007.01266.x 17825075

[31] Meesters S Ossenblok P Colon A Wagner L Schijns O Boon P. Modeling of intracerebral interictal epileptic discharges: evidence for network interactions. Clinic. Neurophysiol. (2018) 129:1276–90. 10.1016/j.clinph.2018.03.021 29679878

[32] Keller CJ Truccolo W Gale JT Eskandar E Thesen T Carlson C. Heterogeneous neuronal firing patterns during interictal epileptiform discharges in the human cortex. Brain. (2010) 133:1668–81. 10.1093/brain/awq112 20511283 PMC2877906

[33] Cuello-Oderiz C Von Ellenrieder N Sankhe R Olivier A Hall J Dubeau F. Value of ictal and interictal epileptiform discharges and high frequency oscillations for delineating the epileptogenic zone in patients with focal cortical dysplasia. Clinic Neurophysiol. (2018) 129:1311–9. 10.1016/j.clinph.2018.02.003 29523391

[34] Hu DK Mower A Shrey DW Lopour BA. Effect of interictal epileptiform discharges on EEG-based functional connectivity networks. Clinic Neurophysiol. (2020) 131:1087–98. 10.1016/j.clinph.2020.02.014 32199397 PMC7784722

[35] Hamer HM Luders HO Knake S Fritsch B Oertel WH Rosenow F. Electrophysiology of focal clonic seizures in humans: a study using subdural and depth electrodes. Brain. (2003) 126:547–55. 10.1093/brain/awg051 12566276

[36] Tyvaert L Hawco C Kobayashi E Levan P Dubeau F Gotman J. Different structures involved during ictal and interictal epileptic activity in malformations of cortical development: an EEG-fMRI study. Brain. (2008) 131:2042–60. 10.1093/brain/awn145 18669486 PMC3792088

[37] Davis KA Devries SP Krieger A Mihaylova T Minecan D Litt B. The effect of increased intracranial EEG sampling rates in clinical practice. Clinic Neurophysiol. (2018) 129:360–7. 10.1016/j.clinph.2017.10.039 29288992 PMC5955774

[38] Shannon CE. Communication in the presence of noise. Proceed IRE. (1949) 37:10–21. 10.1109/JRPROC.1949.232969

[39] Bartolomei F Bettus G Stam CJ Guye M. Interictal network properties in mesial temporal lobe epilepsy: a graph theoretical study from intracerebral recordings. Clinic Neurophysiol. (2013) 124:2345–53. 10.1016/j.clinph.2013.06.003 23810635

[40] Coito A Biethahn S Tepperberg J Carboni M Roelcke U Seeck M. Interictal epileptogenic zone localization in patients with focal epilepsy using electric source imaging and directed functional connectivity from low-density EEG. Epilepsia Open. (2019) 4:281–92. 10.1002/epi4.12318 31168495 PMC6546067

[41] Varotto G Tassi L Franceschetti S Spreafico R Panzica F. Epileptogenic networks of type II focal cortical dysplasia: a stereo-EEG study. Neuroimage. (2012) 61:591–8. 10.1016/j.neuroimage.2012.03.090 22510255

[42] Zweiphenning WJEM Keijzer HM Van Diessen E Klooster MV Van Klink NEC. Increased gamma and decreased fast ripple connections of epileptic tissue: A high-frequency directed network approach. Epilepsia. (2019) 60:1908–20. 10.1111/epi.16296 31329277 PMC6852371

[43] Grobelny BT London D Hill TC North E Dugan P Doyle WK. Betweenness centrality of intracranial electroencephalography networks and surgical epilepsy outcome. Clinic Neurophysiol. (2018) 129:1804–12. 10.1016/j.clinph.2018.02.135 29981955

[44] Lagarde S Roehri N Lambert I Trebuchon A Mcgonigal A Carron R. Interictal stereotactic-EEG functional connectivity in refractory focal epilepsies. Brain. (2018) 141:2966–80. 10.1093V/brain/awy214 30107499

[45] Chvojka J Kudlacek J Chang WC Novak O Tomaska F Otahal J. The role of interictal discharges in ictogenesis-A dynamical perspective. Epilepsy Behav. (2021) 21:121. 10.1016/j.yebeh.2019.106591 31806490

