# Supplementary material for: Brain network analysis of interictal epileptiform discharges from ECoG to identify epileptogenic zone in pediatric patients with epilepsy and focal cortical dysplasia type II: A retrospective study
Source: Front Neurol. 2022 Aug 5;13:901633. doi: 10.3389/fneur.2022.901633 (PMC9388828; doi:10.3389/fneur.2022.901633)
Supplement: Supplementary file 1 [file Data_Sheet_1.docx]

**Brain network analysis of interictal epileptiform discharges from ECoG to identify epileptogenic zone in pediatric patients with epilepsy and focal cortical dysplasia type II: A retrospective study**

**SUPPLEMENTARY MATERIAL**

**TABLE S1** Demographic and clinical data of all ten patients

| **Patient** | **sex** | **Seizure onset age**  **(in years and months)** | **Age at surgery**  **(in years and months)** | **F/U duration**  **(in years and months)** | **Location of resection** | **IED types** | **Outcomes (Engel classification)** |
| --- | --- | --- | --- | --- | --- | --- | --- |
| 1 | M | 8y5m | 13y9m | 7y4m | left F cortisectomy + left sensory cortex | Polyspike–wave complexes | Ⅰ |
| 2 | M | 6y9m | 8y11m | 3y6m | right F cortisectomy | Spike–wave discharges | Ⅰ |
| 3 | M | 4m | 6y3m | 1y8m | right F lobectomy + right insulectomy | Spike–wave discharges | Ⅰ |
| 4 | M | 5y0m | 17y2m | 8y2m | right F cortisectomy + right sensory cortex | Polyspike–wave complexes | Ⅰ |
| 5 | M | 5y0m | 15y3m | 9y2m | left F lobectomy | Spike–wave discharges | Ⅰ |
| 6 | M | 3y0m | 12y3m | 9y2m | right F lobectomy + right T cortisectomy | Spike–wave discharges | Ⅰ |
| 7 | M | 2y0m | 16y9m | 2y1m | left T lobectomy + left insulectomy | Spike–wave discharges | Ⅰ |
| 8 | F | 1m | 5y1m | 1y5m | left T–O lobectomy | Spike–wave discharges | Ⅰ |
| 9 | M | 7y0m | 17y11m | 1y4m | left T cortisectomy + left O cortisectomy | Spike–wave discharges | Ⅱ* |
| 10 | F | 3m | 9y4m | 1y3m | left O lobectomy | Spike–wave discharges | Ⅰ |

F/U, follow–up; F, frontal; T, temporal; O, occipital; IED, interictal epileptiform discharges; *Patient 9 was seizure–free for 8 months.

**TABLE S2** Location and number of ECoG electrodes of all ten patients

| **Patient** | **ECoG location** | **Number of channels** | | | |
| --- | --- | --- | --- | --- | --- |
|  |  | **Total** | **EZ** | **MZ** | **NZ** |
| 1 | Left F, T, P, O | 96 | 5 | 12 | 79 |
| 2 | Right F, T, P, O | 128 | 7 | 10 | 111 |
| 3 | Right F, P, T | 79 | 28 | 12 | 39 |
| 4 | Right F, P, T, O | 126 | 4 | 13 | 109 |
| 5 | Left F | 63 | 20 | 15 | 28 |
| 6 | Right F, T, P | 128 | 57 | 10 | 61 |
| 7 | Left F, T, P | 81 | 24 | 15 | 42 |
| 8 | Left T, P, O | 95 | 51 | 15 | 29 |
| 9 | Left T, P, O | 84 | 19 | 22 | 43 |
| 10 | Left P, O | 95 | 35 | 8 | 52 |

EZ, epileptogenic zone; MZ, margin zone; NZ, normal zone; F, frontal lobe; T, temporal lobe; P, parietal lobe; O, occipital lobe


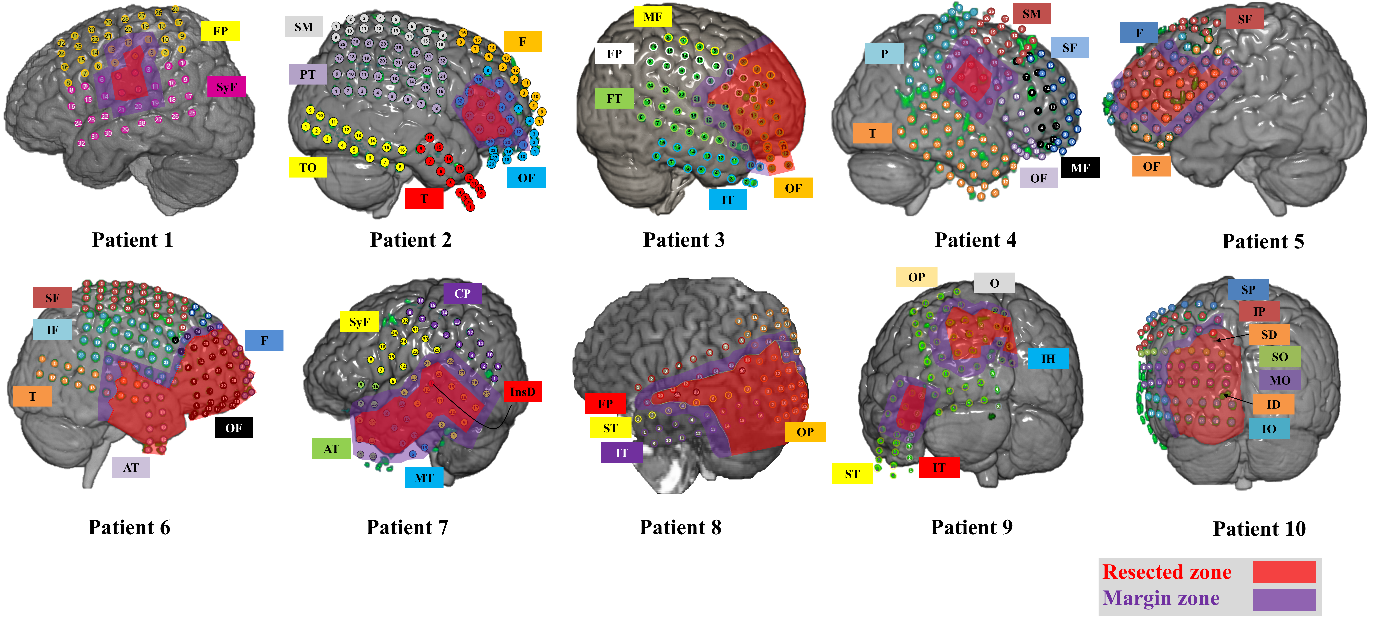


**Figure S1** ECoG implantation with the resected zone (red outlined region) and the margin zone (purple outlined region) for all ten patients. AT, anterior temporal; CP, central parietal; dA, depth A; dB, depth B; dC, depth C; F, frontal; FP, frontal parietal; FT, frontal temporal; ID, inferior depth; IF, inferior frontal; IH, interhemisphere; InsD, insular depth; IO, inferior occipital; IP, inferior parietal; IT, inferior temporal; MF, middle frontal; MO, middle occipital; MT, middle temporal; O, occipital; OF, orbital frontal; OP, occipital parietal; P, parietal; PT, parietal temporal; SD, superior depth; SF, superior frontal; SM, superior motor; SO, superior occipital; SP, superior parietal; ST, superior temporal; SyF, sylvian fissure; T, temporal; TO, temporal occipital.

**Phase transfer entropy**

In information theory, the Shannon entropy $H\left( X \right)$ is a random variable of information or uncertainty, written as the following:

| $H\left( X \right)=-\sum_{i=1}^{N} p\left( x_{i} \right)\log\left\{ p\left( x_{i} \right) \right\}$ | (1) |
| --- | --- |

where $p\left( x \right)$ is the probability that $X=x$.

When two variables $X$ and $Y$ share information, mutual information $MI$ can be defined as the following:

| $MI\left( X;Y \right)=H\left( Y \right)-H\left( Y\vert X \right)=H\left( X \right)-H\left( X\vert Y \right)$ | (2) |
| --- | --- |

where $H\left( X \right)$ and $H\left( Y \right)$ are the marginal entropy, $H\left( Y|X \right)$ and $H\left( X|Y \right)$ are the conditional entropies, and are given as the following:

| $H\left( Y\vert X \right)=H\left( X, Y \right)-H\left( X \right)$ | (3) |
| --- | --- |
| $H\left( X\vert Y \right)=H\left( X, Y \right)-H\left( Y \right)$ | (4) |

Additionally, the entropy of variable $Y$ conditioned on two variables, $X$ and $Z$ can be calculated as the following:

| $H\left( Y\vert X,Z \right)=H\left( X, Y,Z \right)-H\left( X,Z \right)$ | (5) |
| --- | --- |

Accordingly, the conditional mutual information $MI$ is defined as follows:

| $MI\left( X;Y\vert Z \right)=H\left( Y\vert Z \right)-H\left( Y\vert X,Z \right)$ | (6) |
| --- | --- |

Thus, for a given time lag $\delta$, the transfer entropy from $\text{X}_{t-\delta}$ to $\text{Y}_{t}$ can be defined as the following conditional information (Yao and Li, 2020):

| ${TE}_{X\to Y}^{\delta}=MI\left( Y_{t};\text{X}_{t-\delta}\vert\text{Y}_{t-\delta} \right)=H\left( Y_{t}\vert\text{Y}_{t-\delta} \right)-H\left( Y_{t};\text{X}_{t-\delta}\vert\text{Y}_{t-\delta} \right)$ | (7) |
| --- | --- |

where ${TE}_{X\to Y}^{\delta}$ represents the uncertainty at the time point $t$ to predict that $\text{Y}$ has source $\text{X}$, which sends the information at time point $t-\delta$.

Lobier et al. defined the phase transfer entropy as follows (Lobier et al., 2014):

| $P{TE}_{X\to Y}=H\left( \text{θ}_{y}\left( t \right),\text{θ}_{y}\left( t' \right) \right)+H\left( \text{θ}_{y}\left( t' \right),\text{θ}_{x}\left( t' \right) \right)-H\left( \text{θ}_{y}\left( t' \right) \right)-H\left( \text{θ}_{y}\left( t \right),\text{θ}_{y}\left( t' \right),\text{θ}_{x}\left( t' \right) \right)$ | (8) |
| --- | --- |

where $\theta\left( t \right)$ represents the current state at time point $t$ and $\theta\left( t' \right)$ represents the previous state at time point $t-\delta$, and joint entropy and marginal entropy are defined as the following:

| $H\left( \text{θ}_{y}\left( t \right),\text{θ}_{y}\left( t' \right) \right)= -\sum p(\text{θ}_{y}\left( t \right),\text{θ}_{y}\left( t' \right))\log p(\text{θ}_{y}\left( t \right),\text{θ}_{y}\left( t' \right))$ | (9) |
| --- | --- |
| $H\left( \text{θ}_{y}\left( t' \right),\text{θ}_{x}\left( t' \right) \right)=-\sum p(\text{θ}_{y}\left( t^{'} \right),\text{θ}_{x}\left( t^{'} \right))log p(\text{θ}_{y}\left( t^{'} \right),\text{θ}_{x}\left( t^{'} \right))$ | (10) |
| $H\left( \text{θ}_{y}\left( t' \right) \right)=-\sum p(\text{θ}_{y}\left( t' \right))\log p(\text{θ}_{y}\left( t' \right))$ | (11) |
| $H\left( \text{θ}_{y}\left( t \right),\text{θ}_{y}\left( t' \right),\text{θ}_{x}\left( t' \right) \right)=-\sum p(\text{θ}_{y}\left( t \right),\text{θ}_{y}\left( t' \right),\text{θ}_{x}\left( t' \right))\log p(\text{θ}_{y}\left( t \right),\text{θ}_{y}\left( t' \right),\text{θ}_{x}\left( t' \right))$ | (12) |

To calculate the probability density in equations (9)–(12), a phase–space binning method was used (Wilmer et al., 2012;Gomez-Herrero et al., 2015) and a histogram–based function was built by binning the appearances of single, pairs, or triplets of phase data across all trials (Lobier et al., 2014). The bin width was computed using Scott’s choice (Scott, 1992):

| ${BW}_{i}=3.5\sigma_{i}/N^{\frac{1}{3}}$ | (13) |
| --- | --- |

where ${BW}_{i}$ represents the bin width for phase time series $\text{θ}_{i}$, $\sigma_{i}$ is the standard deviation, which was defined by Fisher (Fisher, 1993) as a directional variable. Finally, the probability value of each bin was evaluated by dividing the number of points in that bin by the total number of data points (Lobier et al., 2014).

**References:**

Fisher, N.I. (1993). *Statistical analysis of circular data.* Cambridge England ; New York, NY, USA: Cambridge University Press.

Gomez-Herrero, G., Wu, W., Rutanen, K., Soriano, M.C., Pipa, G., and Vicente, R. (2015). Assessing Coupling Dynamics from an Ensemble of Time Series. *Entropy* 17**,** 1958-1970.

Lobier, M., Siebenhuhner, F., Palva, S., and Palva, J.M. (2014). Phase transfer entropy: a novel phase-based measure for directed connectivity in networks coupled by oscillatory interactions. *Neuroimage* 85 Pt 2**,** 853-872.

Scott, D.W. (1992). *Multivariate density estimation : theory, practice, and visualization.* New York: Wiley.

Wilmer, A., De Lussanet, M., and Lappe, M. (2012). Time-Delayed Mutual Information of the Phase as a Measure of Functional Connectivity. *Plos One* 7.

Yao, C.Z., and Li, H.Y. (2020). Effective Transfer Entropy Approach to Information Flow Among EPU, Investor Sentiment and Stock Market. *Frontiers in Physics* 8.

**Table S3** Mann–Whitney U test (two–tailed) for comparative analysis between EZ, MZ, and NZ for each parameter with different threshold values

| ***p* < 0.05** | | **Threshold** | **CC** | **LE** | **OD** | **OS** | **ID** | **IS** | **BC** |
| --- | --- | --- | --- | --- | --- | --- | --- | --- | --- |
| P1 | EZ–MZ | 30% | 0.96 | 0.99 | 0.006^★^ | 0.006^★^ | 0.81 | 0.81 | 0.16 |
|  |  | 50% | 0.80 | 0.51 | 0.006^★^ | 0.006^★^ | 0.81 | 0.81 | 0.51 |
|  |  | 70% | 0.16 | 0.16 | 0.04^★^ | 0.04^★^ | 0.58 | 0.64 | 0.16 |
|  | EZ–NZ | 30% | 0.005^★^ | 0.004^★^ | <0.001^★★^ | <0.001^★★^ | 0.10 | 0.10 | 0.61 |
|  |  | 50% | <0.001^★★^ | <0.001^★★^ | <0.001^★★^ | <0.001^★★^ | 0.08 | 0.08 | 0.47 |
|  |  | 70% | <0.001^★★^ | <0.001^★★^ | <0.001^★★^ | <0.001^★★^ | 0.54 | 0.54 | 0.89 |
|  | MZ–NZ | 30% | <0.001^★★★^ | <0.001^★★★^ | 0.49 | 0.46 | 0.002**^#^** | 0.002**^#^** | 0.008**^#^** |
|  |  | 50% | <0.001^★★★^ | <0.001^★★★^ | 0.09 | 0.10 | <0.001**^##^** | <0.001**^##^** | 0.04**^#^** |
|  |  | 70% | <0.001^★★★^ | <0.001^★★★^ | <0.001^★★★^ | <0.001^★★★^ | <0.001**^##^** | 0.006**^#^** | 0.04**^#^** |
| P2 | EZ–MZ | 30% | 0.23 | 0.81 | <0.001^★★^ | <0.001^★★^ | 0.96 | 0.96 | 0.10 |
|  |  | 50% | 0.89 | 0.42 | <0.001^★★^ | <0.001^★★^ | 0.81 | 0.81 | 0.10 |
|  |  | 70% | 0.003^★^ | <0.001^★★^ | <0.001^★★^ | <0.001^★★^ | 0.13 | 0.13 | 0.01^★^ |
|  | EZ–NZ | 30% | 0.14 | 0.61 | <0.001^★★★^ | <0.001^★★★^ | 0.63 | 0.63 | 0.01^★^ |
|  |  | 50% | 0.81 | 0.02^★^ | <0.001^★★★^ | <0.001^★★★^ | 0.70 | 0.70 | 0.09 |
|  |  | 70% | <0.001^★★^ | <0.001^★★^ | <0.001^★★^ | <0.001^★★^ | 0.03^★^ | 0.03^★^ | 0.006^★^ |
|  | MZ–NZ | 30% | 0.53 | 0.39 | 0.82 | 0.82 | 0.77 | 0.77 | 0.99 |
|  |  | 50% | 0.26 | 0.14 | 0.70 | 0.70 | 0.70 | 0.79 | 0.34 |
|  |  | 70% | 0.008^★^ | 0.007^★^ | 0.005^★^ | 0.006^★^ | 0.12 | 0.13 | 0.13 |
| P3 | EZ–MZ | 30% | 0.89 | 0.24 | 0.03^★^ | 0.03^★^ | 0.32 | 0.32 | 0.21 |
|  |  | 50% | 0.05^★^ | 0.02^★^ | 0.003^★^ | 0.003^★^ | 0.09 | 0.10 | 0.04^★^ |
|  |  | 70% | <0.001^★★^ | <0.001^★★^ | <0.001^★★^ | <0.001^★★^ | 0.008^★^ | 0.008^★^ | 0.26 |
|  | EZ–NZ | 30% | 0.20 | 0.24 | 0.92 | 0.89 | 0.005^★^ | 0.005^★^ | 0.06 |
|  |  | 50% | 0.91 | 0.98 | 0.63 | 0.75 | 0.002^★^ | 0.002^★^ | 0.22 |
|  |  | 70% | 0.003^★^ | 0.003^★^ | 0.007^★^ | 0.007^★^ | <0.001^★★^ | <0.001^★★^ | 0.91 |
|  | MZ–NZ | 30% | 0.30 | 0.04^#^ | 0.03^#^ | 0.03^#^ | 0.19 | 0.19 | 0.90 |
|  |  | 50% | 0.07 | 0.02^#^ | 0.001^#^ | 0.001^#^ | 0.25 | 0.25 | 0.16 |
|  |  | 70% | 0.11 | 0.11 | 0.04^#^ | 0.03^#^ | 0.71 | 0.66 | 0.26 |
| P4 | EZ–MZ | 30% | 0.006^★^ | 0.003^★^ | 0.78 | 0.78 | 0.13 | 0.13 | 0.99 |
|  |  | 50% | 0.003^★^ | 0.003^★^ | 0.35 | 0.41 | 0.13 | 0.13 | 0.68 |
|  |  | 70% | 0.002^★^ | 0.002^★^ | 0.003^★^ | 0.003^★^ | 0.02**^#^** | 0.022**^#^** | 0.99 |
|  | EZ–NZ | 30% | <0.001^★★^ | <0.001^★★^ | 0.94 | 0.94 | 0.05 | 0.05 | 0.33 |
|  |  | 50% | <0.001^★★^ | <0.001^★★^ | 0.27 | 0.28 | 0.01^#^ | 0.01^#^ | 0.36 |
|  |  | 70% | <0.001^★★^ | <0.001^★★^ | <0.001^★★^ | <0.001^★★^ | 0.003**^#^** | 0.003**^#^** | 0.34 |
|  | MZ–NZ | 30% | 0.03^★^ | 0.03^★^ | 0.63 | 0.64 | 0.38 | 0.38 | 0.08 |
|  |  | 50% | 0.04^★^ | 0.03^★^ | 0.78 | 0.82 | 0.51 | 0.36 | 0.39 |
|  |  | 70% | 0.001^★^ | 0.001^★^ | 0.002^★^ | 0.002^★^ | 0.90 | 0.79 | 0.09 |
| P5 | EZ–MZ | 30% | <0.001^★★★^ | <0.001^★★★^ | 0.003^★^ | 0.005^★^ | 0.005^#^ | 0.005^#^ | 0.04^#^ |
|  |  | 50% | <0.001^★★^ | <0.001^★★^ | <0.001^★★^ | <0.001^★★^ | 0.02^#^ | 0.02^#^ | 0.06 |
|  |  | 70% | <0.001^★★★^ | <0.001^★★★^ | <0.001^★★★^ | <0.001^★★★^ | 0.28 | 0.26 | 0.04**^#^** |
|  | EZ–NZ | 30% | <0.001^★★★^ | <0.001^★★★^ | <0.001^★★★^ | <0.001^★★★^ | <0.001^##^ | <0.001^##^ | <0.001^##^ |
|  |  | 50% | <0.001^★★★^ | <0.001^★★★^ | <0.001^★★★^ | <0.001^★★★^ | <0.001^###^ | <0.001^###^ | 0.01^#^ |
|  |  | 70% | <0.001^★★★^ | <0.001^★★★^ | <0.001^★★★^ | <0.001^★★★^ | 0.23 | 0.30 | 0.003**^#^** |
|  | MZ–NZ | 30% | 0.03^★^ | 0.02^★^ | 0.002^★^ | 0.002^★^ | 0.06 | 0.05 | 0.15 |
|  |  | 50% | 0.01^★^ | 0.01^★^ | 0.08 | 0.04^★^ | 0.16 | 0.11 | 0.76 |
|  |  | 70% | 0.03^★^ | 0.03^★^ | 0.04^★^ | 0.04^★^ | 0.44 | 0.50 | 0.25 |
| P6 | EZ–MZ | 30% | 0.08 | 0.05^★^ | 0.49 | 0.48 | 0.63 | 0.61 | 0.40 |
|  |  | 50% | 0.07 | 0.06 | 0.17 | 0.22 | 0.60 | 0.55 | 0.95 |
|  |  | 70% | 0.01^★^ | 0.01^★^ | 0.02^★^ | 0.02^★^ | 0.16 | 0.17 | 0.34 |
|  | EZ–NZ | 30% | 0.001^★^ | <0.001^★★^ | <0.001^★★★^ | <0.001^★★★^ | 0.002^#^ | 0.002^#^ | 0.12 |
|  |  | 50% | <0.001^★★^ | <0.001^★★^ | <0.001^★★^ | <0.001^★★^ | 0.36 | 0.12 | 0.92 |
|  |  | 70% | <0.001^★★★^ | <0.001^★★★^ | <0.001^★★★^ | <0.001^★★★^ | <0.001^★★★^ | <0.001^★★★^ | 0.82 |
|  | MZ–NZ | 30% | 0.49 | 0.42 | 0.10 | 0.08 | 0.17 | 0.18 | 0.18 |
|  |  | 50% | 0.61 | 0.60 | 0.19 | 0.14 | 0.75 | 0.83 | 0.97 |
|  |  | 70% | 0.45 | 0.47 | 0.46 | 0.43 | 0.04^★^ | 0.05 | 0.31 |
| P7 | EZ–MZ | 30% | 0.03^★^ | 0.02^★^ | 0.59 | 0.68 | 0.18 | 0.19 | 0.60 |
|  |  | 50% | 0.05^★^ | 0.04^★^ | 0.99 | 0.92 | 0.61 | 0.56 | 0.23 |
|  |  | 70% | <0.001^★★^ | <0.001^★★^ | <0.001^★★^ | <0.001^★★^ | 0.04^★^ | 0.05 | 0.79 |
|  | EZ–NZ | 30% | 0.001^★^ | <0.001^★★^ | 0.55 | 0.66 | 0.06 | 0.06 | 0.59 |
|  |  | 50% | 0.001^★^ | <0.001^★★^ | 0.93 | 0.91 | 0.07 | 0.05^#^ | 0.04^#^ |
|  |  | 70% | <0.001^★★^ | <0.001^★★^ | <0.001^★★^ | <0.001^★★^ | 0.27 | 0.27 | 0.42 |
|  | MZ–NZ | 30% | 0.99 | 0.95 | 0.66 | 0.62 | 0.77 | 0.74 | 0.97 |
|  |  | 50% | 0.47 | 0.46 | 0.94 | 0.98 | 0.32 | 0.29 | 0.31 |
|  |  | 70% | 0.75 | 0.75 | 0.70 | 0.67 | 0.49 | 0.47 | 0.70 |
| P8 | EZ–MZ | 30% | 0.001^★^ | <0.001^★★^ | 0.98 | 0.98 | <0.001^##^ | <0.001^##^ | 0.61 |
|  |  | 50% | <0.001^★★^ | <0.001^★★^ | <0.001^★★^ | <0.001^★★^ | 0.003^#^ | 0.003^#^ | 0.33 |
|  |  | 70% | <0.001^★★^ | <0.001^★★^ | <0.001^★★^ | <0.001^★★^ | 0.22 | 0.33 | 0.18 |
|  | EZ–NZ | 30% | <0.001^★★★^ | <0.001^★★★^ | 0.42 | 0.36 | <0.001**^###^** | <0.001**^###^** | 0.002^#^ |
|  |  | 50% | <0.001^★★★^ | <0.001^★★★^ | <0.001^★★★^ | <0.001^★★★^ | <0.001**^###^** | <0.001**^###^** | <0.001**^##^** |
|  |  | 70% | <0.001^★★★^ | <0.001^★★★^ | <0.001^★★★^ | <0.001^★★★^ | 0.08 | 0.13 | <0.001**^##^** |
|  | MZ–NZ | 30% | 0.23 | 0.008^★^ | 0.35 | 0.30 | 0.03**^#^** | 0.04**^#^** | 0.17 |
|  |  | 50% | 0.92 | 0.67 | 0.59 | 0.54 | 0.06 | 0.04^#^ | 0.15 |
|  |  | 70% | 0.75 | 0.77 | 0.84 | 0.82 | 0.99 | 0.96 | 0.17 |
| P9 | EZ–MZ | 30% | 0.13 | 0.11 | 0.02^★^ | 0.02^★^ | 0.21 | 0.21 | 0.92 |
|  |  | 50% | 0.09 | 0.06 | 0.11 | 0.13 | 0.46 | 0.40 | 0.37 |
|  |  | 70% | 0.01^★^ | 0.01^★^ | 0.01^★^ | 0.01^★^ | 0.04^★^ | 0.04^★^ | 0.98 |
|  | EZ–NZ | 30% | 0.02^★^ | 0.01^★^ | 0.01^★^ | 0.01^★^ | 0.29 | 0.28 | 0.85 |
|  |  | 50% | 0.02^★^ | 0.01^★^ | 0.01^★^ | 0.01^★^ | 0.71 | 0.60 | 0.06 |
|  |  | 70% | <0.001^★★^ | <0.001^★★^ | <0.001^★★^ | <0.001^★★^ | 0.02^★^ | 0.02^★^ | 0.40 |
|  | MZ–NZ | 30% | 0.36 | 0.40 | 0.93 | 0.90 | 0.86 | 0.87 | 0.99 |
|  |  | 50% | 0.30 | 0.29 | 0.58 | 0.58 | 0.47 | 0.56 | 0.30 |
|  |  | 70% | 0.21 | 0.22 | 0.24 | 0.24 | 0.28 | 0.28 | 0.35 |
| P10 | EZ–MZ | 30% | 0.99 | 0.12 | 0.005^★^ | 0.005^★^ | 0.60 | 0.56 | 0.99 |
|  |  | 50% | 0.99 | 0.13 | 0.03^★^ | 0.03^★^ | 0.70 | 0.84 | 0.93 |
|  |  | 70% | <0.001^★★^ | <0.001^★★^ | 0.001^★^ | 0.001^★^ | 0.003^★^ | 0.004^★^ | 0.82 |
|  | EZ–NZ | 30% | 0.15 | 0.02^★^ | <0.001^★★^ | <0.001^★★^ | 0.55 | 0.58 | 0.68 |
|  |  | 50% | 0.02^★^ | <0.001^★★^ | 0.16 | 0.12 | 0.09 | 0.12 | 0.12 |
|  |  | 70% | <0.001^★★★^ | <0.001^★★★^ | <0.001^★★★^ | <0.001^★★★^ | <0.001^★★★^ | <0.001^★★★^ | 0.23 |
|  | MZ–NZ | 30% | 0.61 | 0.94 | 0.11 | 0.11 | 0.34 | 0.33 | 0.76 |
|  |  | 50% | 0.25 | 0.33 | 0.006^#^ | 0.007^#^ | 0.54 | 0.55 | 0.66 |
|  |  | 70% | 0.38 | 0.41 | 0.97 | 0.92 | 0.21 | 0.22 | 0.22 |

P1, patient 1; EZ, epileptogenic zone; MZ, margin zone; NZ, normal zone; BC, betweenness centrality; CC, clustering coefficient; LE, local efficiency; OD, out–degree; OS, out–strength; ID, in–degree; IS, in–strength; ★ represents *p* < 0.05 with EZ > MZ, EZ > NZ, or MZ > NZ; ★★ represents *p* < 0.001 with EZ > MZ, EZ > NZ, or MZ > NZ; ★★★ represents *p* < 0.00001 with EZ > MZ, EZ > NZ, or MZ > NZ; **#** represents *p* < 0.05 with EZ < MZ, EZ < NZ, or MZ < NZ; **##** represents *p* < 0.001 with EZ < MZ, EZ < NZ, or MZ < NZ.


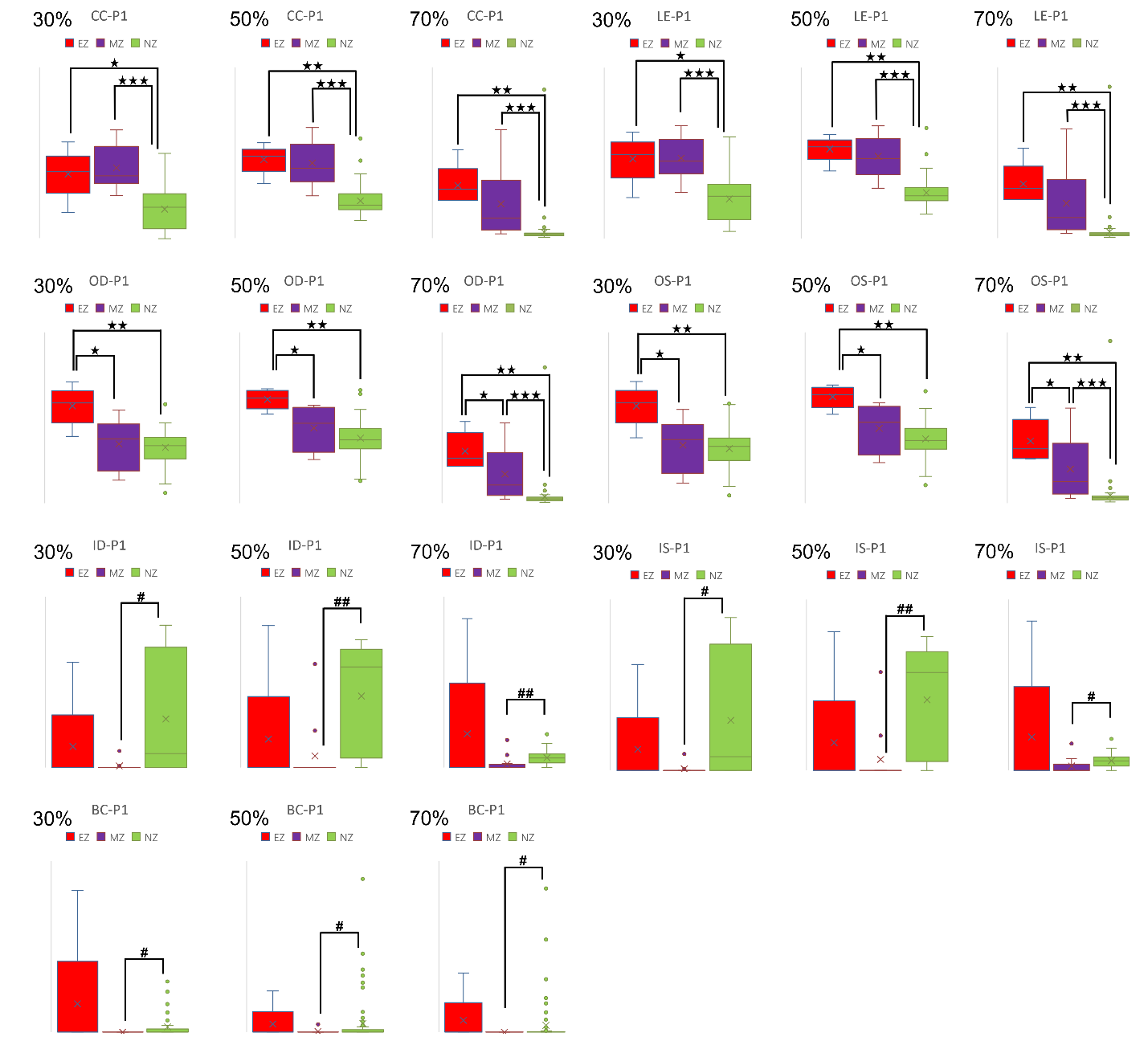


**Figure S2** Box plot of comparative results of all parameters of patient 1 for EZ, MZ, and NZ with 30%, 50%, and 70% threshold. (P1 represents patient 1; ★ represents *p* < 0.05 with EZ > MZ, EZ > NZ, or MZ > NZ; ★★ represents *p* < 0.001 with EZ > MZ, EZ > NZ, or MZ > NZ; ★★★ represents *p* < 0.00001 with EZ > MZ, EZ > NZ, or MZ > NZ; Mann–Whitney U test [two–tailed]).

CC, clustering coefficient; LE, local efficiency; OD, out–degree; OS, out–strength; ID, in–degree; IS, in–strength; BC, betweenness centrality; EZ, epileptogenic zone; MZ, margin zone; NZ, normal zone.


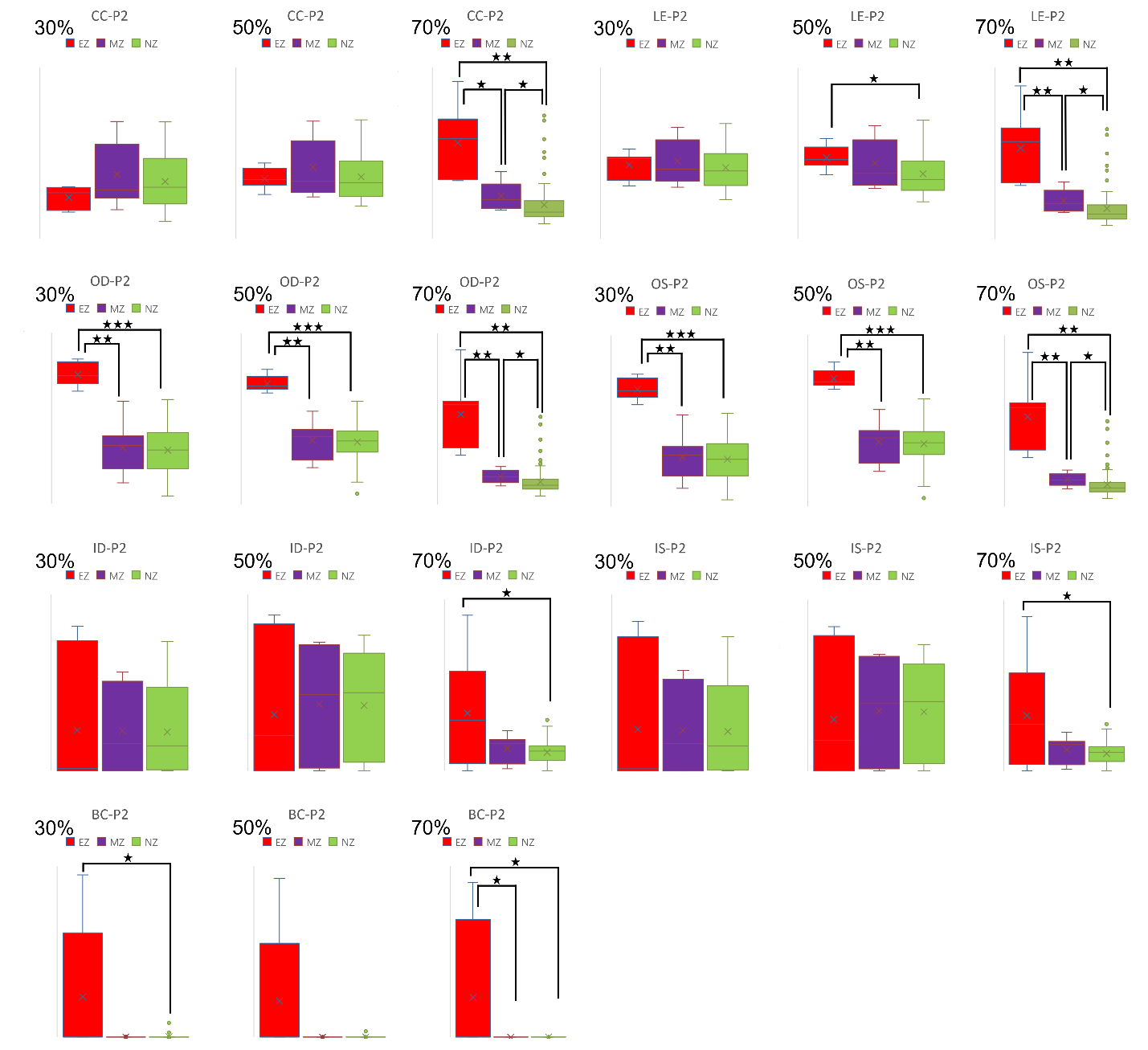


**Figure S3** Box plot of comparative results of all parameters of patient 2 for EZ, MZ, and NZ with 30%, 50%, and 70% threshold. (P2 represents patient 2; ★ represents *p* < 0.05 with EZ > MZ, EZ > NZ, or MZ > NZ; ★★ represents *p* < 0.001 with EZ > MZ, EZ > NZ, or MZ > NZ; ★★★ represents *p* < 0.00001 with EZ > MZ, EZ > NZ, or MZ > NZ; Mann–Whitney U test [two–tailed]).

CC, clustering coefficient; LE, local efficiency; OD, out–degree; OS, out–strength; ID, in–degree; IS, in–strength; BC, betweenness centrality; EZ, epileptogenic zone; MZ, margin zone; NZ, normal zone.


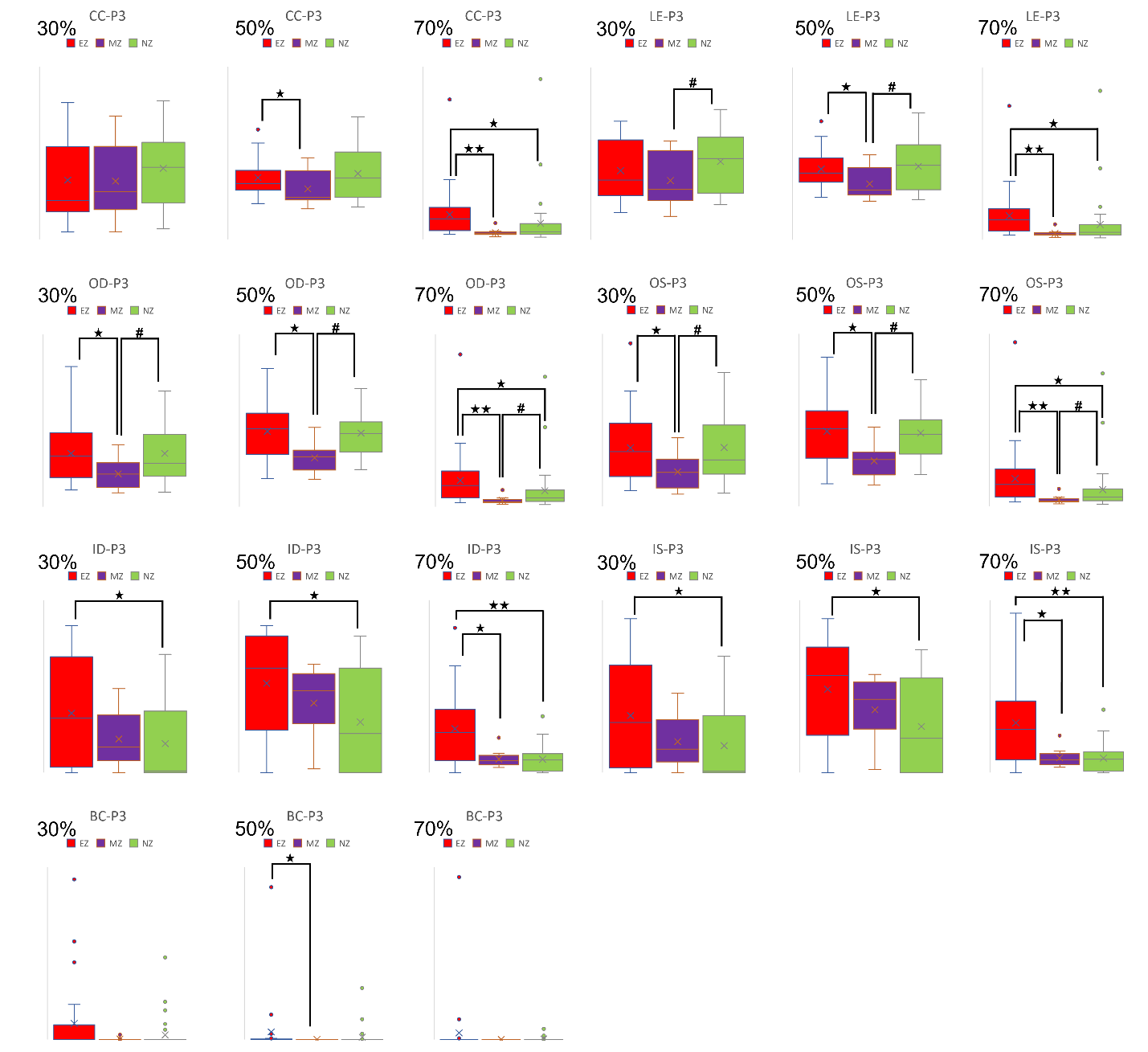


**Figure S4** Box plot of comparative results of all parameters of patient 3 for EZ, MZ, and NZ with 30%, 50%, and 70% threshold. (P3 represents patient 3; ★ represents *p* < 0.05 with EZ > MZ, EZ > NZ, or MZ > NZ; ★★ represents *p* < 0.001 with EZ > MZ, EZ > NZ, or MZ > NZ; ★★★ represents *p* < 0.00001 with EZ > MZ, EZ > NZ, or MZ > NZ; Mann–Whitney U test [two–tailed]).

CC, clustering coefficient; LE, local efficiency; OD, out–degree; OS, out–strength; ID, in–degree; IS, in–strength; BC, betweenness centrality; EZ, epileptogenic zone; MZ, margin zone; NZ, normal zone.


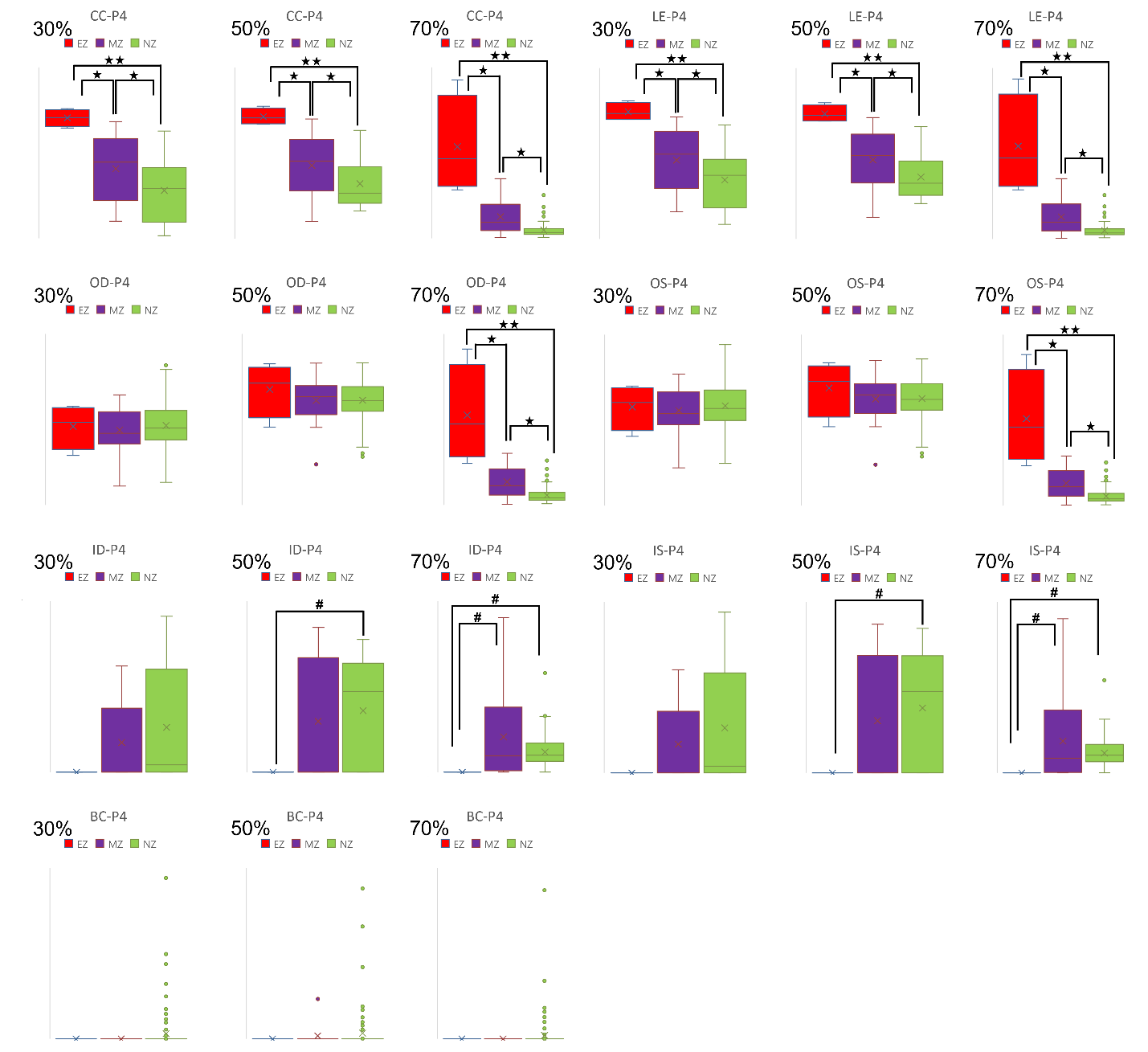


**Figure S5** Box plot of comparative results of all parameters of patient 4 for EZ, MZ, and NZ with 30%, 50%, and 70% threshold. (P4 represents patient 4; ★ represents *p* < 0.05 with EZ > MZ, EZ > NZ, or MZ > NZ; ★★ represents *p* < 0.001 with EZ > MZ, EZ > NZ, or MZ > NZ; ★★★ represents *p* < 0.00001 with EZ > MZ, EZ > NZ, or MZ > NZ; Mann–Whitney U test [two–tailed]).

CC, clustering coefficient; LE, local efficiency; OD, out–degree; OS, out–strength; ID, in–degree; IS, in–strength; BC, betweenness centrality; EZ, epileptogenic zone; MZ, margin zone; NZ, normal zone.


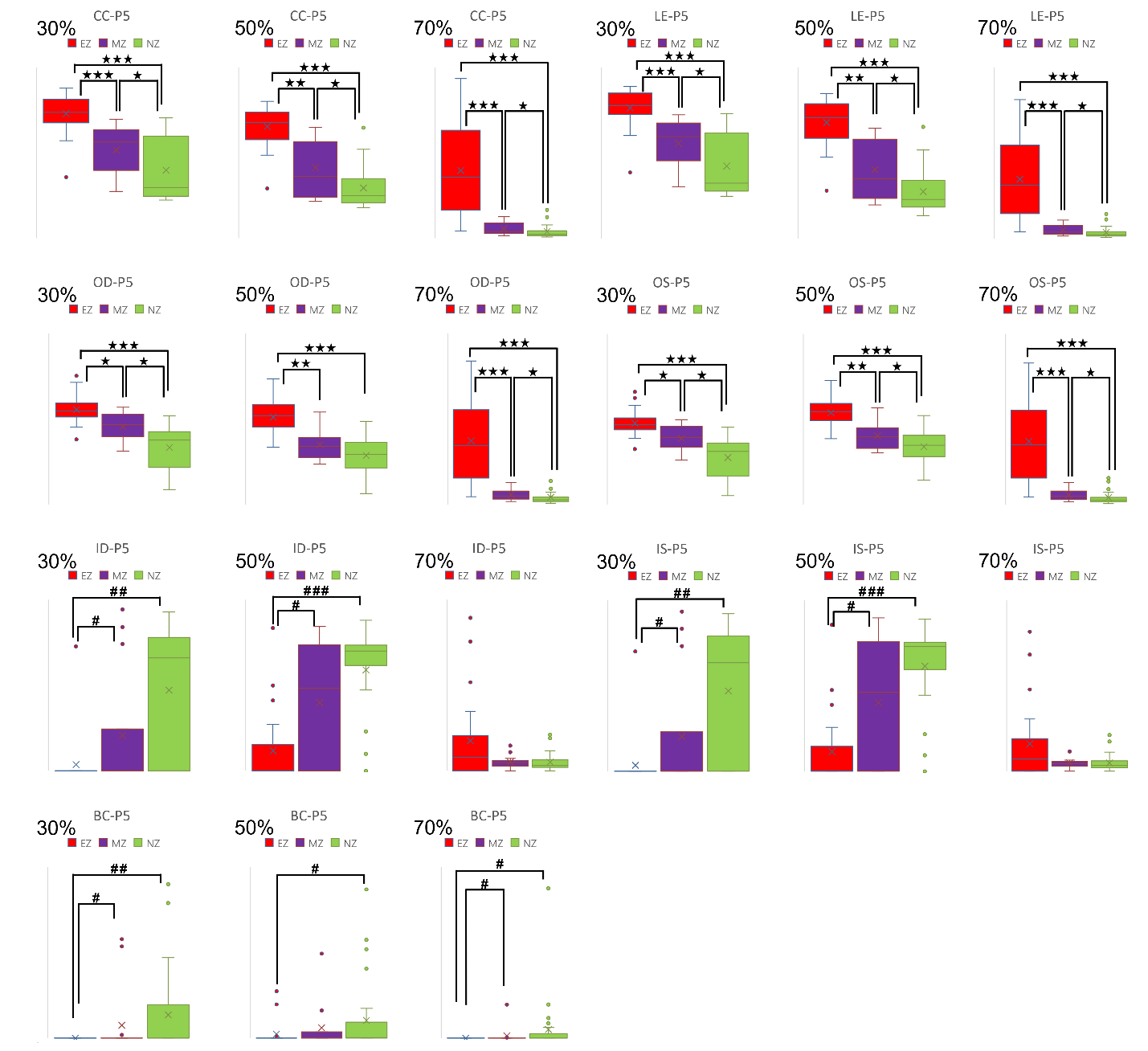


**Figure S6** Box plot of comparative results of all parameters of patient 5 for EZ, MZ, and NZ with 30%, 50%, and 70% threshold. (P5 represents patient 5; ★ represents *p* < 0.05 with EZ > MZ, EZ > NZ, or MZ > NZ; ★★ represents *p* < 0.001 with EZ > MZ, EZ > NZ, or MZ > NZ; ★★★ represents *p* < 0.00001 with EZ > MZ, EZ > NZ, or MZ > NZ; Mann–Whitney U test [two–tailed]).

CC, clustering coefficient; LE, local efficiency; OD, out–degree; OS, out–strength; ID, in–degree; IS, in–strength; BC, betweenness centrality; EZ, epileptogenic zone; MZ, margin zone; NZ, normal zone.


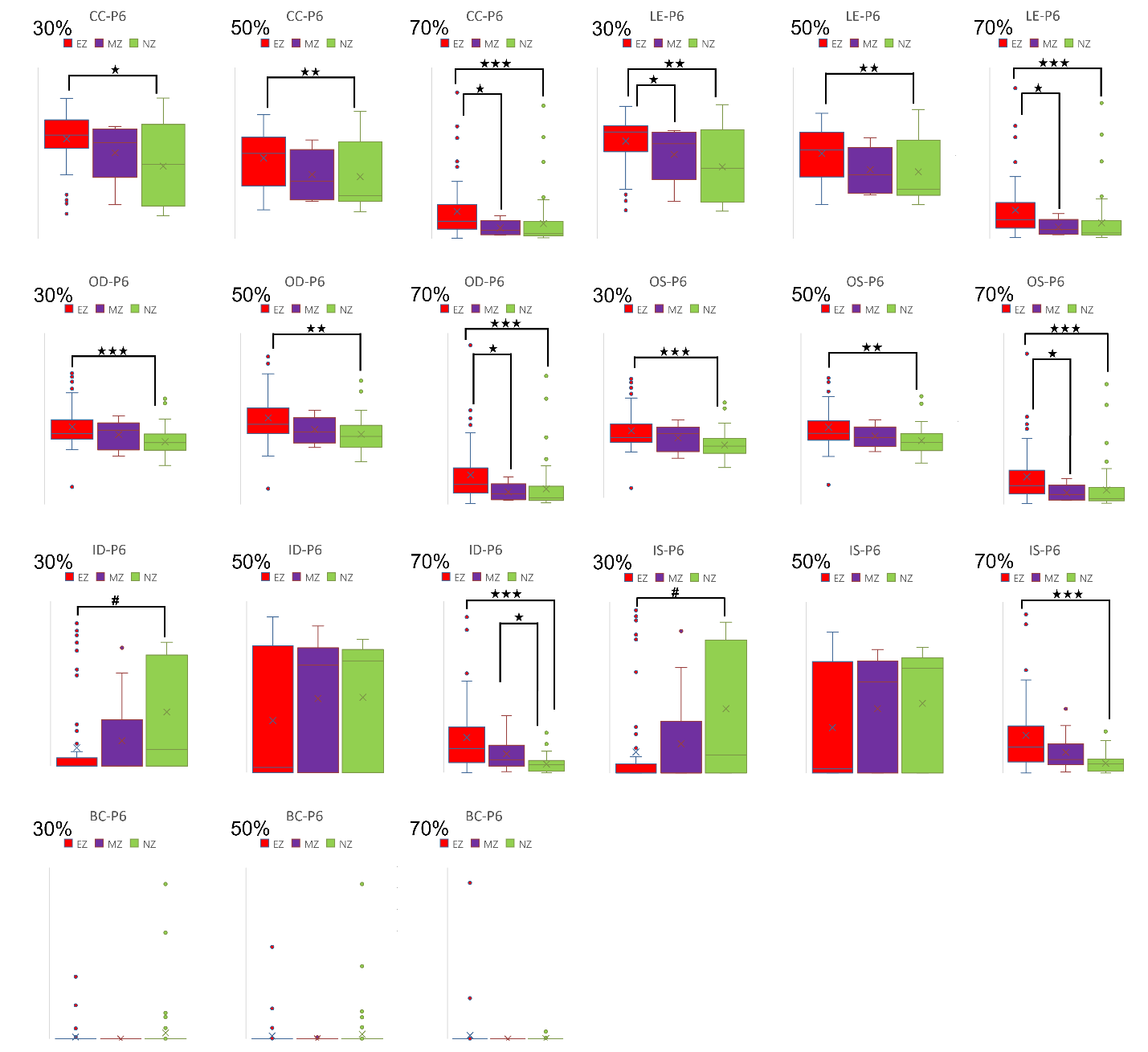


**Figure S7** Box plot of comparative results of all parameters of patient 6 for EZ, MZ, and NZ with 30%, 50%, and 70% threshold. (P6 represents patient 6; ★ represents *p* < 0.05 with EZ > MZ, EZ > NZ, or MZ > NZ; ★★ represents *p* < 0.001 with EZ > MZ, EZ > NZ, or MZ > NZ; ★★★ represents *p* < 0.00001 with EZ > MZ, EZ > NZ, or MZ > NZ; Mann–Whitney U test [two–tailed]).

CC, clustering coefficient; LE, local efficiency; OD, out–degree; OS, out–strength; ID, in–degree; IS, in–strength; BC, betweenness centrality; EZ, epileptogenic zone; MZ, margin zone; NZ, normal zone.


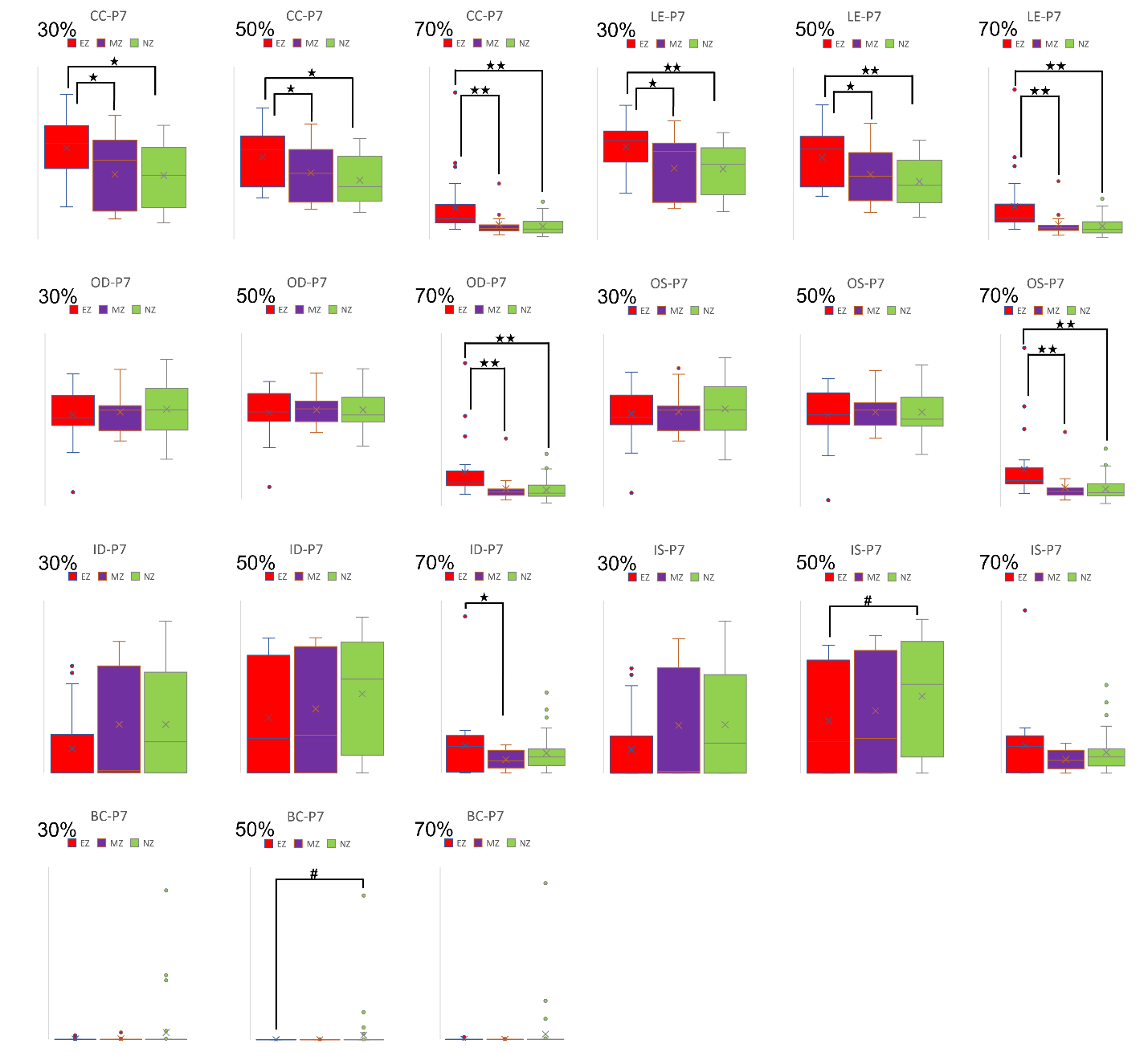


**Figure S8** Box plot of comparative results of all parameters of patient 7 for EZ, MZ, and NZ with 30%, 50%, and 70% threshold. (P7 represents patient 7; ★ represents *p* < 0.05 with EZ > MZ, EZ > NZ, or MZ > NZ; ★★ represents *p* < 0.001 with EZ > MZ, EZ > NZ, or MZ > NZ; ★★★ represents *p* < 0.00001 with EZ > MZ, EZ > NZ, or MZ > NZ; Mann–Whitney U test [two–tailed]).

CC, clustering coefficient; LE, local efficiency; OD, out–degree; OS, out–strength; ID, in–degree; IS, in–strength; BC, betweenness centrality; EZ, epileptogenic zone; MZ, margin zone; NZ, normal zone.


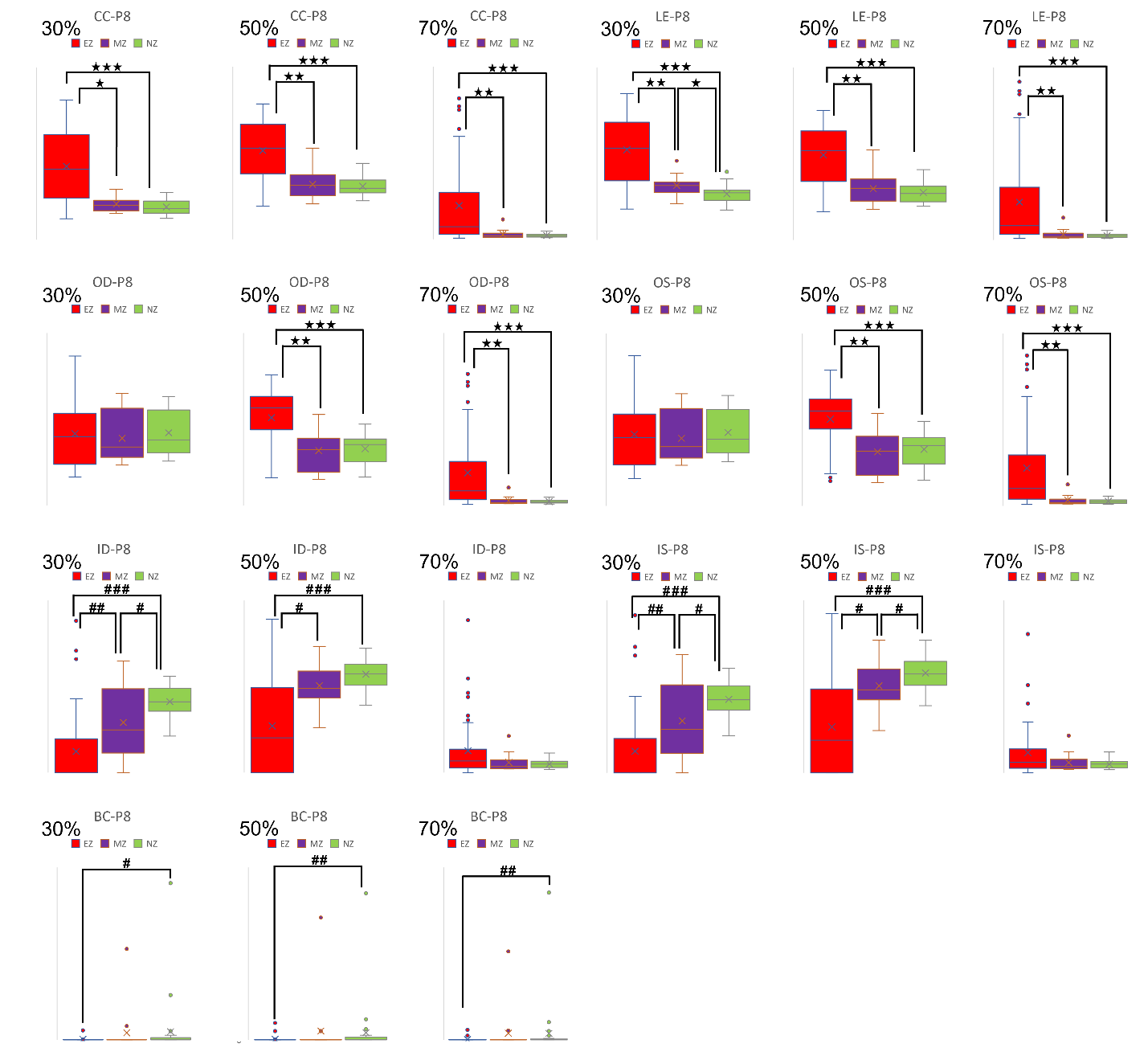


**Figure S9** Box plot of comparative results of all parameters of patient 8 for EZ, MZ, and NZ with 30%, 50%, and 70% threshold. (P8 represents patient 8; ★ represents *p* < 0.05 with EZ > MZ, EZ > NZ, or MZ > NZ; ★★ represents *p* < 0.001 with EZ > MZ, EZ > NZ, or MZ > NZ; ★★★ represents *p* < 0.00001 with EZ > MZ, EZ > NZ, or MZ > NZ; Mann–Whitney U test [two–tailed]).

CC, clustering coefficient; LE, local efficiency; OD, out–degree; OS, out–strength; ID, in–degree; IS, in–strength; BC, betweenness centrality; EZ, epileptogenic zone; MZ, margin zone; NZ, normal zone.


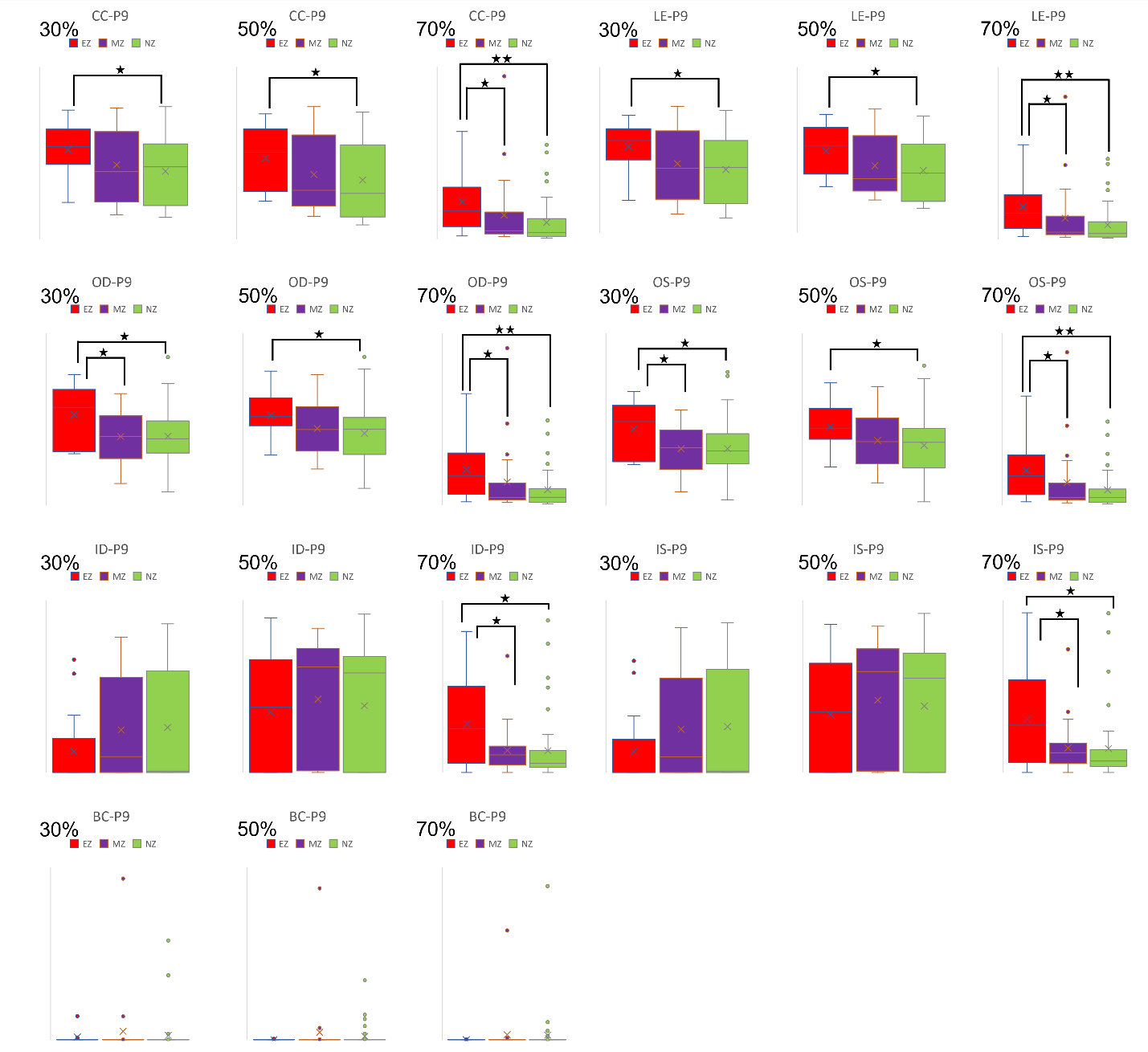


**Figure S10** Box plot of comparative results of all parameters of patient 9 for EZ, MZ, and NZ with 30%, 50%, and 70% threshold. (P9 represents patient 9; ★ represents *p* < 0.05 with EZ > MZ, EZ > NZ, or MZ > NZ; ★★ represents *p* < 0.001 with EZ > MZ, EZ > NZ, or MZ > NZ; ★★★ represents *p* < 0.00001 with EZ > MZ, EZ > NZ, or MZ > NZ; Mann–Whitney U test [two–tailed]).

CC, clustering coefficient; LE, local efficiency; OD, out–degree; OS, out–strength; ID, in–degree; IS, in–strength; BC, betweenness centrality; EZ, epileptogenic zone; MZ, margin zone; NZ, normal zone.


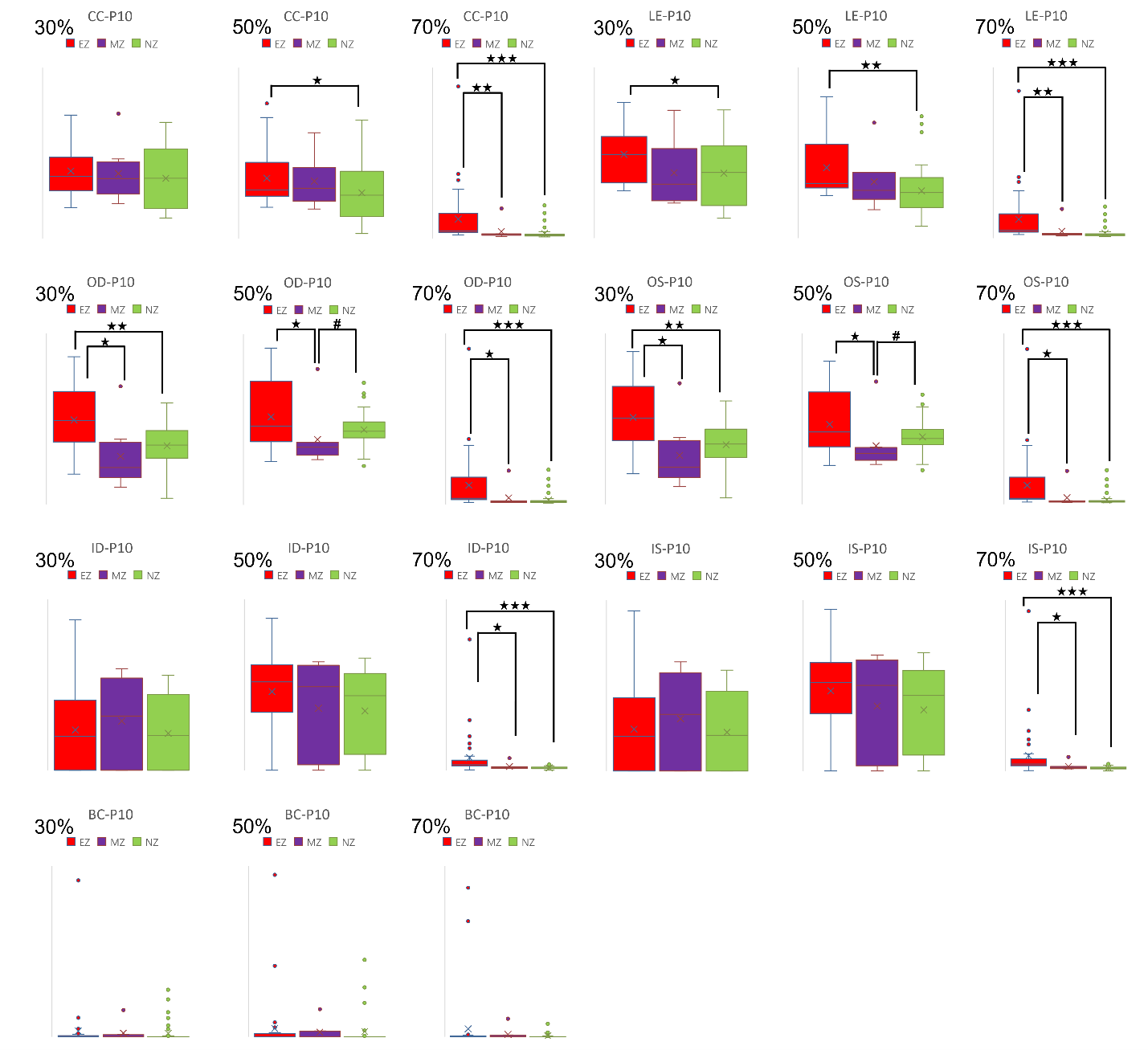


**Figure S11** Box plot of comparative results of all parameters of patient 10 for EZ, MZ, and NZ with 30%, 50%, and 70% threshold. (P10 represents patient 10; ★ represents *p* < 0.05 with EZ > MZ, EZ > NZ, or MZ > NZ; ★★ represents *p* < 0.001 with EZ > MZ, EZ > NZ, or MZ > NZ; ★★★ represents *p* < 0.00001 with EZ > MZ, EZ > NZ, or MZ > NZ; Mann–Whitney U test [two–tailed]).

CC, clustering coefficient; LE, local efficiency; OD, out–degree; OS, out–strength; ID, in–degree; IS, in–strength; BC, betweenness centrality; EZ, epileptogenic zone; MZ, margin zone; NZ, normal zone.

**Table S4** Accuracy of predictive results of EZ vs MZ and EZ vs NZ using logistic regression

| **(Probability > 0.5 = 1; else = 0)** | **EZ (1) vs MZ (0)** | **EZ (1) vs NZ (0)** |
| --- | --- | --- |
| P1 | 0% vs 100% | 80% vs 98.68% |
| P2 | 57.14% vs 100% | 57.14% vs 98.20% |
| P3 | 82.14% vs 66.67% | 71.43% vs 87.18% |
| P4 | 0% vs 100% | 75% vs 98.17% |
| P5 | 85% vs 86.67% | 80% vs 89.29% |
| P6 | 100% vs 0% | 71.93% vs 90.16% |
| P7 | 87.5% vs 73.33% | 83.33% vs 92.86% |
| P8 | 82.35% vs 60% | 80.39% vs 89.66% |
| P9 | 63.16% vs 81.82% | 52.63% vs 90.32% |
| P10 | 97.14% vs 75% | 80% vs 94.23% |

P1, patient 1; EZ, epileptogenic zone; MZ, margin zone; NZ, normal zone.


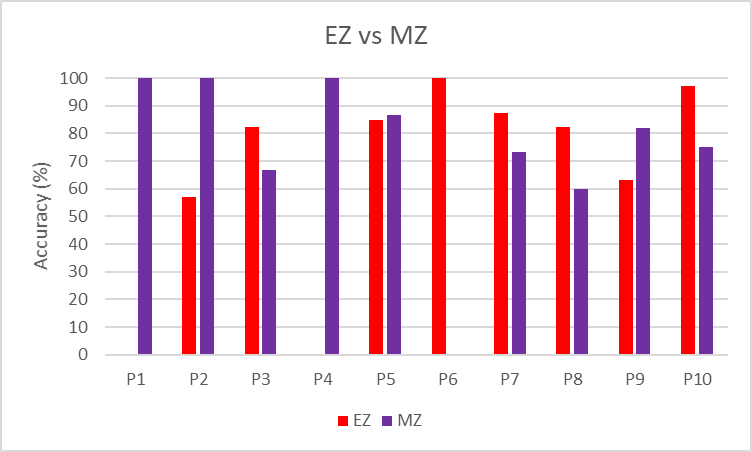


**Figure S12** Histogram plot of the accuracy of predictive results of EZ vs MZ using logistic regression. (EZ, epileptogenic zone; MZ, margin zone.)


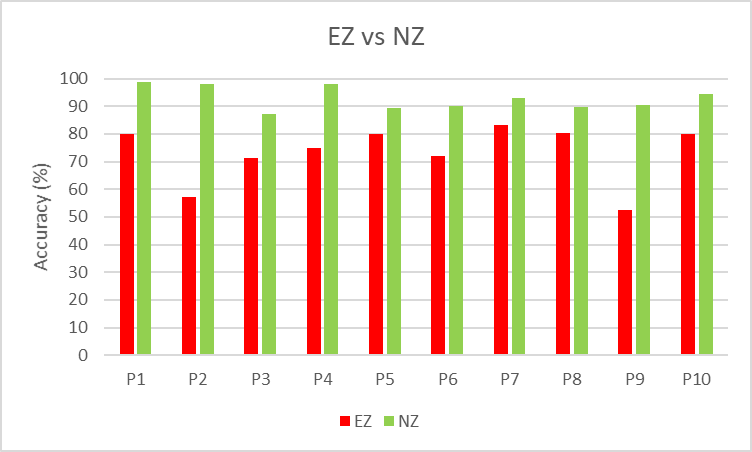


**Figure S13** Histogram plot of the accuracy of predictive results of EZ vs NZ using logistic regression. (EZ, epileptogenic zone; NZ, normal zone.)
